# Model of the pathway of −1 frameshifting: Long pausing

**DOI:** 10.1016/j.bbrep.2016.01.017

**Published:** 2016-01-29

**Authors:** Ping Xie

**Affiliations:** Key Laboratory of Soft Matter Physics and Beijing National Laboratory for Condensed Matter Physics, Institute of Physics, Chinese Academy of Sciences, Beijing 100190, China

**Keywords:** Ribosome, Translational regulation, Translation elongation, Protein synthesis, Virus

## Abstract

It has been characterized that the programmed ribosomal −1 frameshifting often occurs at the slippery sequence on the presence of a downstream mRNA pseudoknot. In some prokaryotic cases such as the *dnaX* gene of *Escherichia coli*, an additional stimulatory signal—an upstream, internal Shine–Dalgarno (SD) sequence—is also necessary to stimulate the efficient −1 frameshifting. However, the molecular and physical mechanism of the −1 frameshifting is poorly understood. Here, we propose a model of the pathway of the −1 translational frameshifting during ribosome translation of the *dnaX* −1 frameshift mRNA. With the model, the single-molecule fluorescence data (Chen et al. (2014) [29]) on the dynamics of the shunt either to long pausing or to normal translation, the tRNA transit and sampling dynamics in the long-paused rotated state, the EF-G sampling dynamics, the mean rotated-state lifetimes, etc., are explained quantitatively. Moreover, the model is also consistent with the experimental data (Yan et al. (2015) [30]) on translocation excursions and broad branching of frameshifting pathways. In addition, we present some predicted results, which can be easily tested by future optical trapping experiments.

## Highlights

• A model of −1 FS is proposed.
• Long pausing associated with −1 FS is explained and studied.
• tRNA transit and sampling dynamics in the long-paused rotated state is studied.
• EF-G sampling dynamics in the long-paused rotated state is studied.
• Mean rotated-state lifetimes are studied.

## 1 Introduction

Programmed ribosomal frameshifting is a process where specific signals in the messenger RNA (mRNA) direct the translating ribosome to shift the reading frame. Many viruses including HIV-1 employ the programmed ribosomal frameshifting to control the ratio between structural and enzymatic proteins [1], [2], [3], [4], [5], [6], [7], [8], [9], [10], [11]. When the reading frame is shifted by one base in the 3′ direction it is called +1 frameshifting, while when the reading frame is shifted by one base in the 5′ direction it is called −1 frameshifting [8], [9], [10], [11]. It was characterized that the amino acid starvation, i.e., the limitation for particular amino acids, can induce the +1 frameshifting [8], [9], [10], [11], [12], [13]. The −1 frameshifting often occurs at the slippery sequence with a form of X_XXY_YYZ on the presence of a downstream mRNA pseudoknot, where codons are shown in the initiation reading frame, with dashes separating in-frame triplets [1], [2], [3], [4], [5], [6], [7], [8], [9], [10], [11], [14], [15], [16], [17]. In some prokaryotic cases, an additional stimulatory signal—an upstream, internal Shine–Dalgarno (SD) sequence—is also necessary to stimulate the efficient −1 frameshifting. For example, the *dnaX* gene of *Escherichia coli* has three stimulatory signals—an SD sequence, an A_AAA_AAG slippery sequence and a downstream stem loop or hairpin [11], [17], [18], [19], [20], [21].

To understand how the −1 frameshifting is stimulated at the slippery sequence on the presence of the downstream mRNA pseudoknot, several models have been proposed. Leger et al. [22] proposed that the −1 frameshifting occurs when deacylated transfer RNA (tRNA) is located in the E/E site, the peptidyl-tRNA located in the P/P site and the aminoacyl-tRNA located in the A/T entry site. Plant et al. [23] proposed a 9 Å model, which is based on the proposal of a movement of 9 Å by the anticodon loop of the aminoacyl-tRNA between the A/T state of initial binding and the A/A state of the full accommodation. The movement induces the −1 frameshifting. Based on the cryo-EM imaging of the mammalian 80S ribosome stalled at an mRNA pseudoknot structure which contains a structurally distorted tRNA in A/P′ hybrid state [24], [25]. Namy et al. proposed that the stereochemical mismatch between the pseudoknot structure and the entrance geometry of the mRNA channel blocks the entry of the downstream mRNA, causing a tension in the mRNA that bends the tRNA in a (+) sense (3′) direction. As a result, the codon-anticodon interaction breaks over the slippery sequence, allowing a spring-like relaxation of the tRNA in a (−) sense (5′) direction and thus causing the tRNA to re-pair with the mRNA in the −1 position. Based on the above three models, Liao et al. [26] developed a kinetic model which proposes that the −1 frameshifting can occur during the translocation step, as proposed in the model by Leger et al. [22] and that by Namy et al. [24], and/or the aminoacyl-tRNA accommodation step, as proposed in the model by Plant et al. [23]. Recently, Bailey et al. [27] developed a mathematical model that treats mRNA translation and associated −1 frameshifting as a stochastic process in which the transition probabilities are based on the energetics of local molecular interactions, with which the location and efficiency of −1 frameshift events in HIV-1 were studied. We proposed another model for the dynamics of the −1 frameshifting during the translation of the mRNA containing the slippery sequence and downstream mRNA pseudoknot [28], where a systematical analysis of the −1 frameshifting that can occur during every transition step in the elongation phase showed that the −1 frameshifting takes place mainly during the translocation over the sequence, as proposed by Namy et al. [24], and simultaneously provided a consistent explanation of a lot of available independent experimental data.

More recently, Chen et al. [29] made a detailed experimental study of the dynamics of the −1 frameshifting during translation of the *dnaX* −1 frameshift mRNA by using single-molecule fluorescence to track directly the compositional and conformational dynamics of individual ribosomes at each codon. Based on their observations they proposed a model of dynamic pathway of the *dnaX* −1 frameshifting (see Fig. S1). In the model, the stochastic interaction of the ribosome with the hairpin helix in an open or closed state and/or formation of the SD and antiSD pairing interaction represent the shunt to either pausing in the rotated state or normal translation. If the hairpin is in open state, the translocation of the ribosomes with the normal translation is coupled with the reverse intersubunit rotation. If the hairpin is in closed state, the translocation of the paused ribosomes is uncoupled with the reverse intersubunit rotation (called “uncoupled” translocation), causing the ribosomes to enter into the long-paused rotated state. However, a quantitative calculation showed that the opening probability of the hairpin helix is only 5.5×10^−4^ (see Section S1), which significantly deviates from the observation showing that about 25% fraction of the ribosomes undergo the normal translation [29]. Then, what is the molecular mechanism of the hairpin and the SD and inti-SD interaction stimulating the shunt to either the long-paused rotated state or the normal translation? Moreover, as the “uncoupled” translocation involves no reverse intersubunit rotation, then how the “uncoupled” translocation occurs is unclear. In the long-paused rotated state, the detailed mechanism of how elongation factor G (EF-G) and tRNA compete for the binding to the ribosome and how the rotated state is finally resolved by EF-G, etc., is also not clear.

In this work, we propose an alternative model of the −1 frameshifting during translation of the *dnaX* −1 frameshift mRNA, addressing the above-mentioned unclear issues and making quantitative explanations of various experimental data observed by Chen et al. [29]. The model can also explain the more recent experimental data of Yan et al. [30] on translocation excursions and broad branching of frameshifting pathways. For simplicity, in the model we do not consider the forward rotation of the 30S head, which will be considered in the next work [31] to explain the biochemical data of Caliskan et al. [32] on the kinetics of EF-G binding and dissociation and on the kinetics of movement of tRNAs inside the ribosome.

## 2 Models

In order to understand easily the pathway of the −1 translational frameshifting during ribosome translation of the *dnaX* −1 frameshift mRNA used in the single-molecule experiments of Chen et al. [29], we firstly present the model of the elongation pathway for the simplest case of translation through the single-stranded mRNA and then present the model for the more complicated case of translation through the duplex region of mRNA. We base mainly on the following arguments and pieces of experimental evidence to build up the models of the translation elongation by the ribosome.

(i) The available structural data [33] showed that the binding of tRNA to the A-site of the ribosome results in the 30S ribosomal subunit to transit from an open to a closed form, in which the shoulder and head domains are rotated towards the subunit center, whereas when the decoding center is unoccupied the 30S subunit is in the open conformation. Based on these structural data, we argue that the ternary complex consisting of the aminoacyl-tRNA, EF-Tu and GTP can bind efficiently to the ribosome with an open conformation of the 30S subunit, whereas the ternary complex cannot bind efficiently to the ribosome with the closed form of the 30S subunit. After the binding of the ternary complex, the subsequent codon recognition results in the transition of the 30S subunit from the open to closed conformation.
(ii) The peptidyl transfer or removal from the P-site tRNA results in the ribosome in a “labile” state, allowing the relative rotation between the two ribosomal subunits, with one conformation as non-rotated and the other one as rotated [34], [35], [36], [37], [38], [39]. The labile ribosome also allows the binding of EF-G. The binding of EF-G complexed with either GTP [35], [36], [39] or GDP [40], [41] facilitates transition to and then stabilizes the rotated state.
(iii) We argue that in the closed conformation of the 30S subunit, the binding of EF-G.GTP to the rotated state and then GTP hydrolysis drives a conformational rearrangement of the ribosome (referred to as ribosomal unlocking, as done in the literature), inducing the 30S subunit to transit from the closed to open conformation, opening the mRNA channel, and then facilitating the reverse intersubunit rotation from the rotated to non-rotated state and Pi release. By contrast, in the open conformation of the 30S subunit, the binding of EF-G.GTP and then GTP hydrolysis is inefficient to induce the ribosomal unlocking. The argument is inferred from the following pieces of experimental evidence. It is experimentally shown that a tRNA anticodon stem-loop bound to the 30S A site is minimally required for translocation of mRNA [42], and the translocation can only occur efficiently after the ribosomal unlocking catalyzed by GTP hydrolysis [43]. On the other hand, the presence of the tRNA anticodon stem-loop bound to the 30S A site implies that the codon recognition has occurred, resulting in the 30S subunit to transit to closed conformation [33].
(iv) The 50S E and P sites have a high affinity for deacylated tRNA and the peptidyl-tRNA, respectively [44], [45].
(v) We argue that after transition to the non-rotated conformation the mRNA channel in the 30S subunit becomes tight, as proposed before based on available structural data [46], [47]. In other words, in the non-rotated conformation the mRNA channel is always tight.
(vi) We argue that the peptidyl-tRNA in the “canonical” P/P conformation induces efficiently the labile ribosome to be “non-labile”,^1^ as evidenced from the cryo-EM studies [36]. The ribosome in the non-labile state inhibits the intersubunit rotations and prohibits the binding of EF-G. In other words, the peptidyl-tRNA in the “non-canonical” P/P conformation such as the severely deformed peptidyl-tRNA cannot induce efficiently the labile ribosome to be non-labile.

### 2.1 Model of the elongation pathway of translation of the single-stranded mRNA

**Fig. 1:**
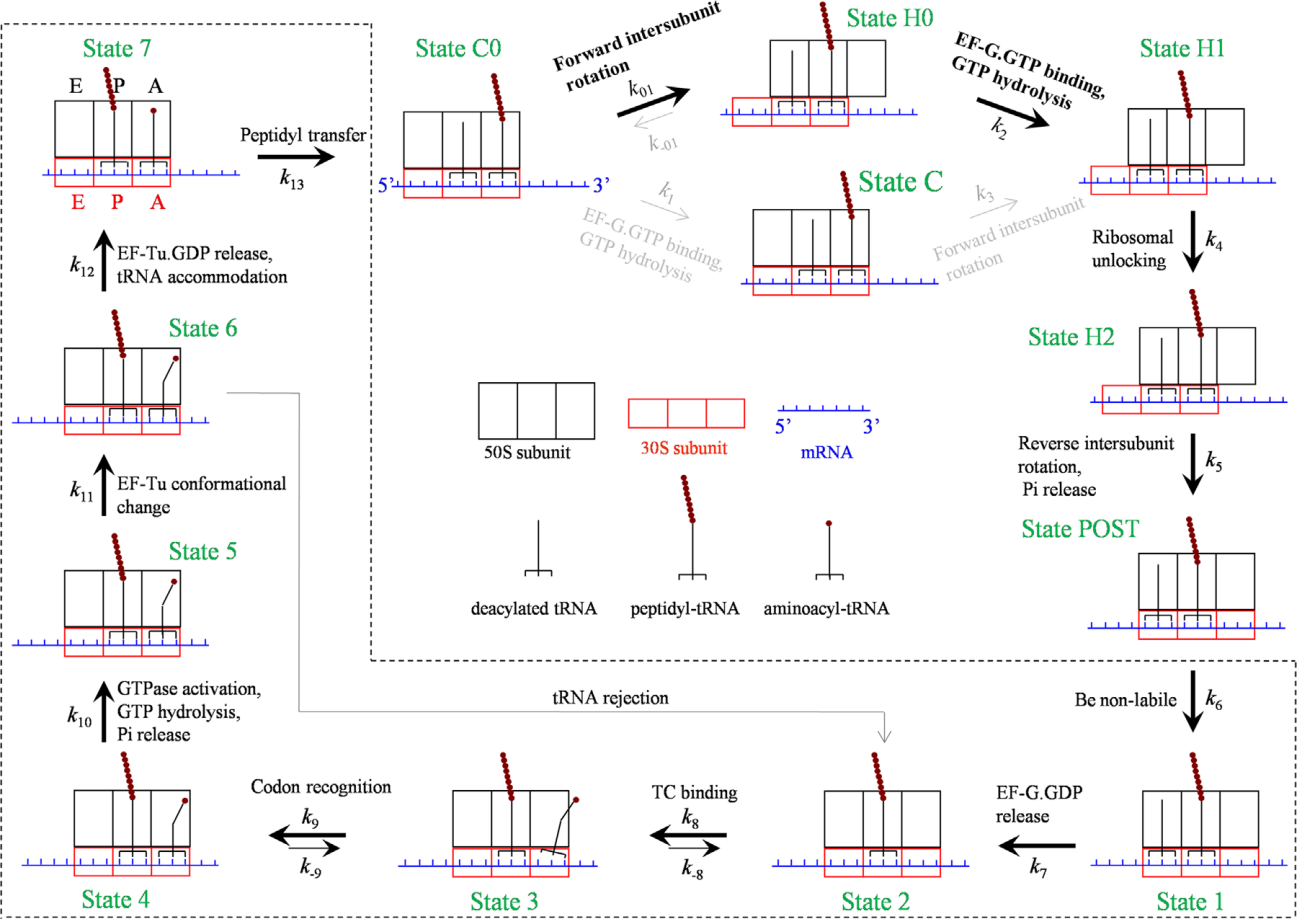
Schematic representation of the elongation pathway for ribosome translation through the single-stranded mRNA (see text for detailed description).

The model of the elongation pathway at low concentration of EF-G.GTP is shown in Fig. 1 [48]. We begin with just after the peptidyl transfer, with deacylated tRNA in the P/P site and the peptidyl-tRNA in the A/A site (State C0). Before EF-G.GTP binding, the labile ribosome allows the spontaneous counterclockwise (viewed from the exterior of the 30S) rotation of the 30S subunit relative to the 50S subunit and vice verse, which are called forward and reverse intersubunit rotations, respectively. The spontaneous intersubunit rotations result in the ribosomal complex to transit from the non-rotated State C0 to rotated or hybrid State H0 and vice verse. The two states are in thermodynamic equilibrium, with the majority being in State H0 [37], [38], [39]. Since in both State C0 and State H0 the 30S A site is occupied, the binding of the ternary complex is not allowed. EF-G.GTP can bind to the labile State C0 and State H0 [49], [50], [51], [52]. (i) If EF-G.GTP binds to State H0 (becoming State H1), after rapid GTP hydrolysis the ribosomal unlocking occurs, opening the mRNA channel and the 30S subunit (State H2). The subsequent reverse intersubunit rotation makes the 30S subunit move downstream relative to the mRNA that is coupled with the two tRNAs by one codon, while the high affinity of the 50S E and P sites for the two tRNAs [44], [45] fixes the two tRNAs to the 50S subunit. This results in the transition of State H2 to posttranslocation state (State POST). Facilitated by the ribosomal unlocking, Pi is also released rapidly and independently of the reverse intersubunit rotation [43]. (ii) If EF-G.GTP binds to State C0 (becoming State C), the transition from State C to State H1 is facilitated mildly [39] and the hybrid State H1 is then stabilized [35], [36], [39]. Since the EF-G-facilitated transition from State C to State H1 (with a rate <10 s^−1^ [39], [53]) is much slower than GTP hydrolysis (with a rate of about 250 s^−1^ [43]), GTP hydrolysis occurs mainly at State C and State H1 is in EF-G.GDP.Pi form, consistent with the available experimental data [54]. In State H1 the ribosomal unlocking occurs (becoming State H2). Then, the reverse intersubunit rotation results in the transition of State H2 to State POST.

In State POST, the peptidyl-tRNA in “canonical” P/P conformation induces the ribosome to be non-labile (State 1), accelerating EF-G.GDP release (State 2). Since the non-labile ribosome prohibits the binding of EF-G.GTP, only the ternary complex can now bind efficiently to the ribosome with an open 30S subunit in the partially bound T/A state (State 3). The subsequent codon recognition (State 4) induces closing of the 30S subunit and triggers GTPase activation, GTP hydrolysis and Pi release (State 5), resulting in a large-scale conformational change of EF-Tu to the GDP-bound form (State 6). EF-Tu.GDP is then released and the aminoacyl-tRNA is accommodated into its fully bound A/A state (State 7). Then the peptidyl transfer leads to State C0, from which the next elongation cycle will proceed.

### 2.2 Model of the elongation pathway of translation through the mRNA duplex at non-slippery sites

**Fig. 2:**
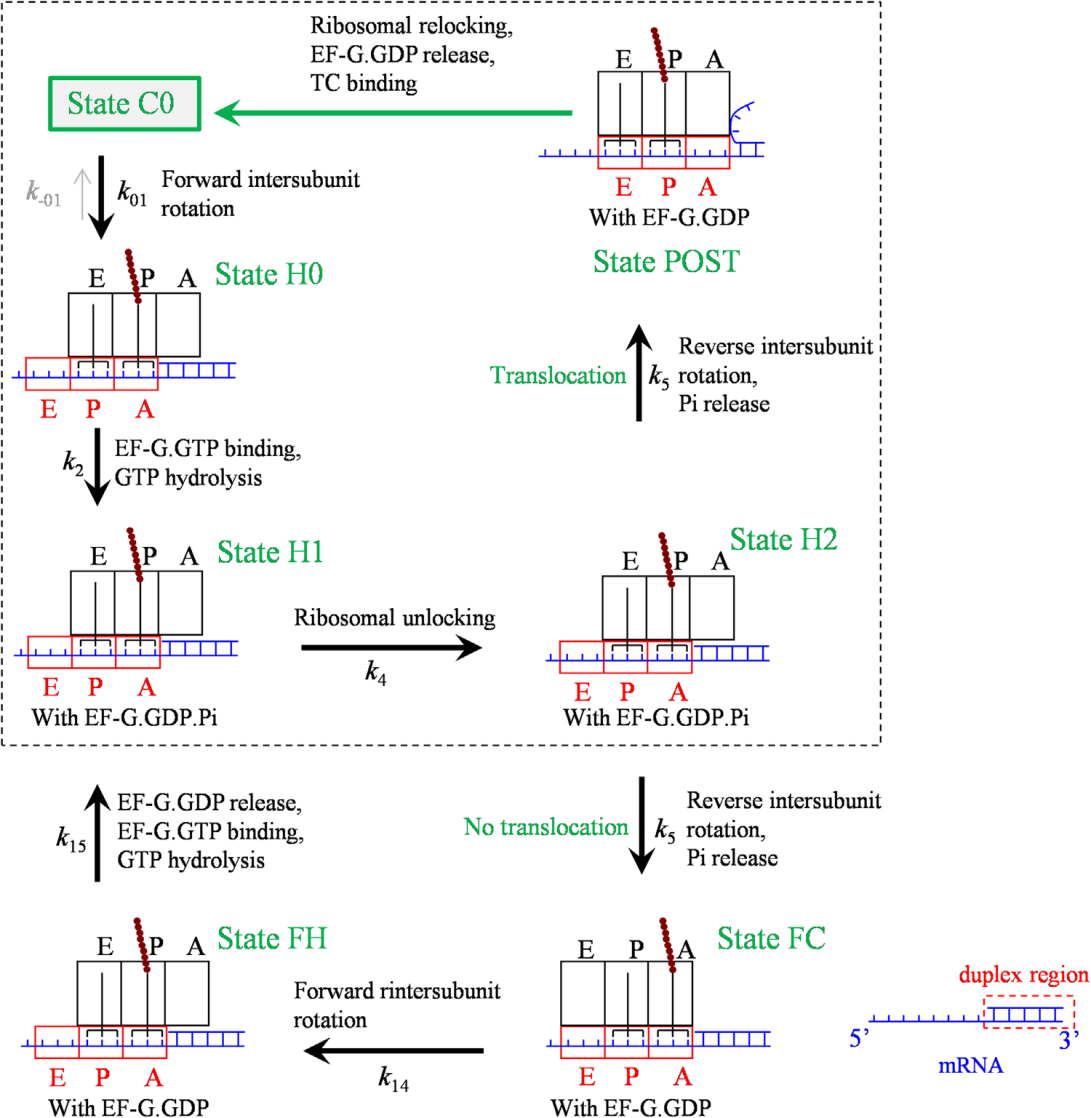
Schematic representation of the elongation pathway for ribosome translation through the mRNA duplex at non-slippery sites (see text for detailed description). Note that the transitions inside the box correspond to the transitions in Fig. 1.

In Fig. 1, after the ribosomal unlocking (State H2) no resistance is present to impede the downstream movement of the 30S subunit along the mRNA. Thus, with the high affinity of the 50S E and P sites fixing the two tRNAs to the 50S subunit, the subsequent reverse intersubunit rotation makes the 30S subunit move downstream relative to the mRNA that is coupled with the two tRNAs by one codon with a 100% probability. However, if the downstream duplex is present, due to the competition between the free energy change of unwinding three mRNA base pairs and that of breaking the interaction of the two tRNAs with the 50S E and P sites, two possibilities of transitions from State H2 can be caused by the reverse intersubunit rotation (Fig. 2) [55]. (i) The reverse intersubunit rotation makes the 30S subunit move downstream relative to the mRNA coupled with the two tRNAs by overcoming the free energy of unwinding three mRNA base pairs, while the high binding energy of the two tRNAs to the 50S E and P sites fixes the two tRNAs to the 50S subunit. This results in the transition of State H2 to State POST, as in Fig. 1, which is called effective translocation. (ii) The reverse intersubunit rotation makes the 50S subunit move relative to the two tRNAs by overcoming the binding energy of the two tRNAs to the 50S E and P sites, while the resistance from the downstream duplex prevents the 30S subunit from moving relative to the mRNA coupled with the two tRNAs. This results in the transition of State H2 to the classical non-rotated pretranslocation state (called futile state, denoted by State FC), which is called futile translocation.

In the non-rotated conformation (State FC), the mRNA channel becomes tight. Since the peptidyl-tRNA is not in the P/P site, the ribosome cannot become non-labile. EF-G.GDP then facilitates State FC transiting to the rotated/hybrid state (State FH). After EF-G.GDP release, EF-G.GTP binding and then GTP hydrolysis, State FH becomes State H1, from which the transition to State H2 continues. After transition to State POST, the transitions to State C0 (indicated by the green arrow) are the same as those (inside the region bounded by broken lines) in Fig. 1.

The peculiarity of the model (Fig. 2) is the occurrence of the futile translocation, with the presence of State FC and State FH. State FC and State FH have similar structures to State C and State H1, respectively. The differences between them are that State FC and State FH are bound with EF-G.GDP while State C and State H1 are bound with EF-G.GTP or EF-G.GDP.Pi. In the model (Fig. 2), the ribosome uses only one mechanism to unwind the mRNA duplex [55], rather than uses two mechanisms as proposed by Qu et al. [56]. As shown before, with the consideration of the futile translocation various single-molecule experimental data such as the optical-trapping data on the rate and dwell-time distribution of translation through the mRNA duplex versus the pulling force to unzip the duplex [56], [57] and the smFRET (single-molecule fluorescence resonance energy transfer) data on the dynamics of slow dissociation of deacylated tRNA from the E site induced by the downstream secondary structures [58] can be explained quantitatively [55], [59], [60]. In particular, the recent high-resolution smFRET data of Kim et al. [61] give a strong support to the futile translocation, which showed that when translates through the mRNA containing the downstream stem loop the ribosomal complex exhibits multiple fluctuations between the classical non-rotated state (State FC in Fig. 2) and hybrid state (State FH, State H1 and State H2) before undergoing mRNA translocation (State POST) at saturating EF-G, whereas when translates through the mRNA lacking the stem loop the ribosomal complex samples the hybrid state (State H1 and State H2) only once before undergoing mRNA translocation [62]. In addition, the consideration of the futile translocation is consistent with the proposal of futile elongation factor 2 (eEF2) cycling during ribosomal translocation in 80S ribosome with the presence of mRNA secondary structures by Flanagan et al. [25].

### 2.3 Model of the elongation pathway of translation through the mRNA duplex at the slippery site

**Fig. 3:**
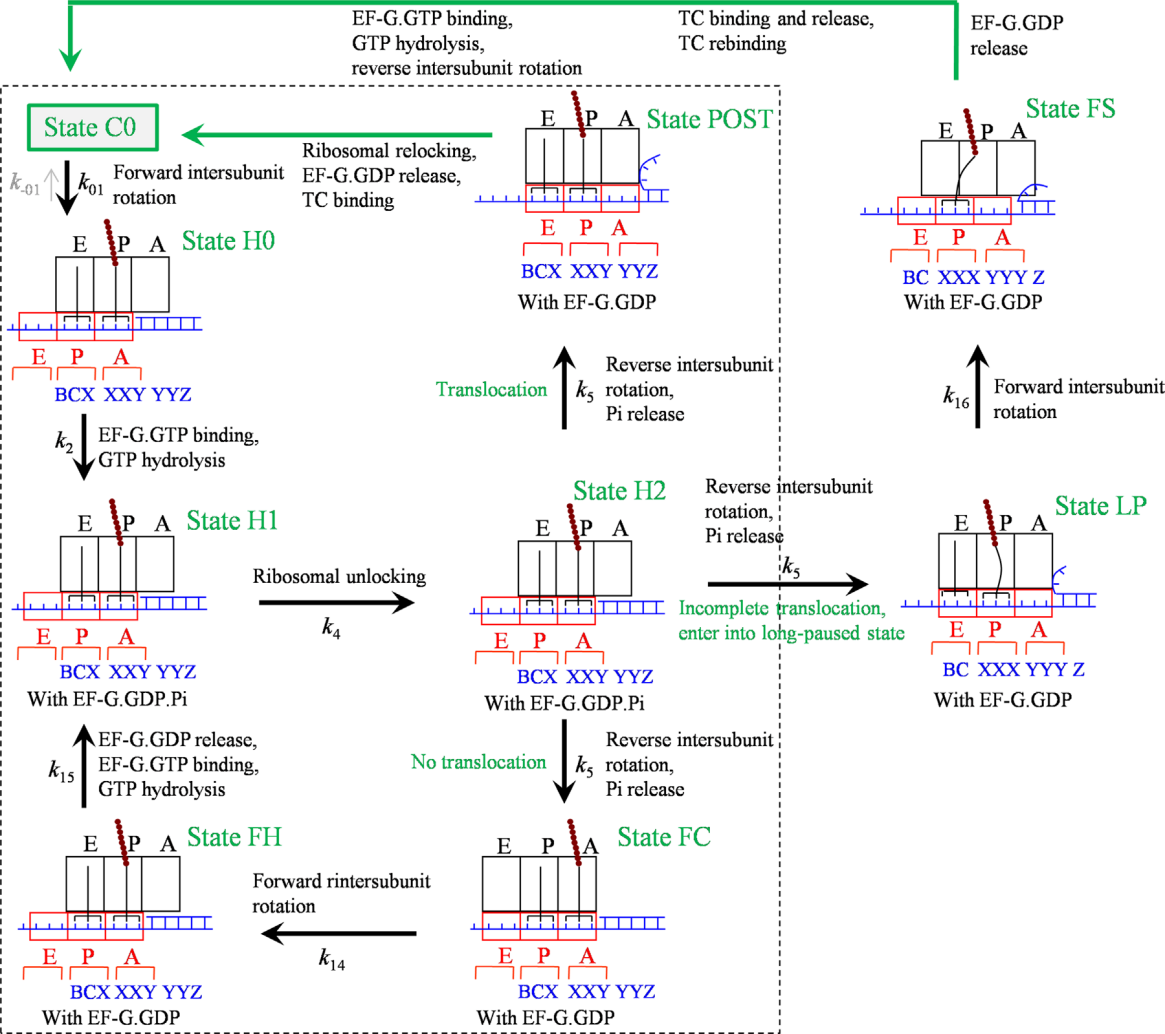
Schematic representation of the elongation pathway for ribosome translation through the mRNA duplex at the slippery site (see text for detailed description). The transitions from State FS to State C0 are shown in Fig. 4. The transitions from State NF to State C0 are similar to the transitions from State FS to State C0. Note that the transitions inside the box correspond to the transitions in Fig. 2.

As shown in Fig. 2, at the non-slippery site, after the ribosomal unlocking the reverse intersubunit rotation can result in the transition of State H2 either to State POST or to State FC. At the slippery site, besides these two transitions the reverse intersubunit rotation can also result in both the movement of the 30S subunit relative to the mRNA by unwinding only two mRNA base pairs and shifting the anticodon of the peptidyl-tRNA from pairing with codon XXY to pairing with codon XXX and the movement of the 50S subunit relative to the mRNA by bending the peptidyl-tRNA, due to the specific affinity of the peptidyl moiety for the 50S P site [45] and the interaction of the anticodon of peptidyl-tRNA with codon XXX (Fig. 3).^2^ This causes the transition of State H2 to State LP (called long-paused state). In contrast to the transitions to State POST and to State FC, where the reverse intersubunit rotation is realized by the movement of the 30S subunit relative to the tRNA–mRNA complex with the 50S subunit being fixed to the tRNAs and by the movement of the 50S subunit relative to the tRNAs with the 30S subunit being fixed to the tRNA–mRNA complex, respectively, in the transition to State LP the reverse intersubunit rotation is realized by the movements of both the 30S and 50S subunits relative to the mRNA.

If transition to State POST or to State FC occurs, the transitions from State POST to State C0 or the transitions from State FC to State H1 are the same as those in Fig. 2, with the transitions in Fig. 2 corresponding to the transitions inside the box of Fig. 3. If transition to State LP occurs, with the non-rotated conformation the mRNA channel becomes tight. But, the severely bent peptidyl-tRNA, which is not in the “canonical” P/P conformation, cannot efficiently induce the ribosome to be non-labile. EF-G.GDP facilitates the ribosome transiting to the rotated state (State FS) and then stabilizes the rotated state. Since in State LP the anticodon of the deacylated tRNA is not paired with the 30S E site codon, the deacylated tRNA would be dissociated readily.

The peculiarity of the model (Fig. 3) is the existence of State LP and State FS bound with EF-G.GDP, where the peptidyl-tRNA adopts the bent conformation, as proposed by Namy et al. [24], [25] based on their structural observations. In State FS, due to the peptidyl moiety being fixed to the 50S P site by the specific interaction between them, the movement of 50S subunit relative to the mRNA that is coupled to the anticodon of tRNA causes the bending of tRNA. In the case with only downstream mRNA secondary structure but without upstream SD sequence, considering that the pulling on the mRNA that arises from the annealing tendency of the unwound mRNA base pairs would also cause the anticodon stem loop of the tRNA to bias toward the A-site, the structural feature of State FS would be similar to that observed in 80S ribosomal complex bound with eEF2 by Namy et al. [24], [25] showing a spring-like deformation of the tRNA in A/P′ state. For the system with no SD sequence involvement, although the bent tRNA is biased toward the A-site, the A-site is left open to some extent, allowing for aminoacyl-tRNA binding. Thus, the model (Fig. 3) could also be applicable to the system with the IBV pseudoknot but without the SD sequence [24], [25].

#### 2.3.1 The pathway of transitions from state FS to state C0

**Fig. 4:**
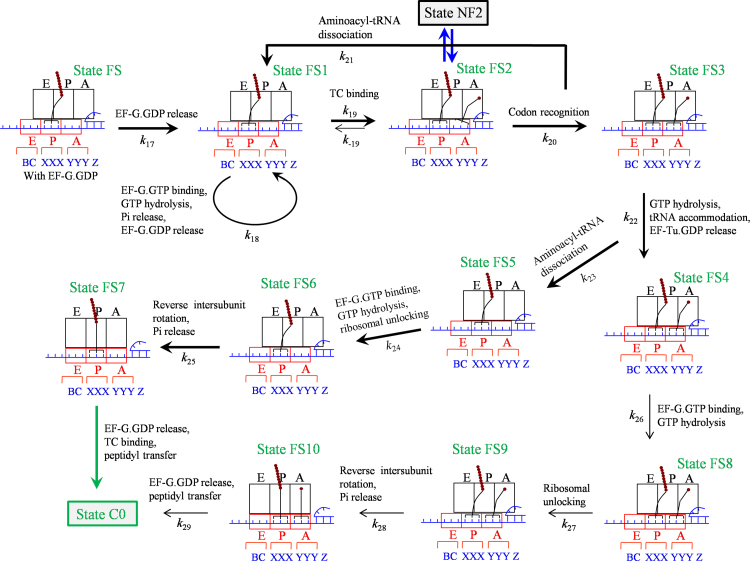
Schematic representation of the pathway after entering into the long-paused rotated state (see text for detailed description). State FS is the same as that shown in Fig. 3.

From State FS, the pathway of transitions to State C0 is shown in Fig. 4. After EF-G.GDP release, since the ribosome in State FS1 is in labile state EF-G.GTP can bind, and since the 30S subunit is in the open conformation the ternary complex can also bind efficiently. Thus, both EF-G.GTP and the ternary complex compete for the binding to State FS1, which is consistent with the single-molecule data [29]. First, consider EF-G.GTP binding. In the open conformation of the 30S subunit, the binding of EF-G.GTP and then GTP hydrolysis is inefficient to induce the ribosomal unlocking. Without ribosomal unlocking facilitating the reverse intersubunit rotation, the ribosome is kept in the rotated conformation during the reaction cycle of EF-G.GTP binding, GTP hydrolysis, Pi release and then EF-G.GDP release.

Then, consider the binding of the ternary complex (State FS2). If the arriving tRNA is cognate to codon YYZ, the codon recognition will facilitate the peptidyl-tRNA to shift the reading frame from codon XXX (State FS2) to codon XXY (State NF2). If the arriving tRNA is cognate to codon YYY, no shift of the reading frame of the peptidyl-tRNA will occur. Thus, whether the −1 frameshifting can occur or not is determined mainly by the arriving tRNAs in State FS2, which is in turn determined by their magnitudes in the binding affinity to the ribosome and the relative concentrations in solution. The codon recognition induces the open 30S subunit transiting to closed conformation, triggering GTPase activation, GTP hydrolysis and Pi release (State FS3). The resultant conformational change of EF-Tu induces the accommodation of the aminoacyl-tRNA and EF-Tu.GDP release (State FS4). Note that during the codon recognition, i.e., before the 30S subunit becoming closed, the aminoacyl-tRNA can be dissociated [63], [64], returning to State FS1. Note also that after the codon recognition, i.e., after the 30S subunit becoming closed, but before the completion of the accommodation, the aminoacyl-tRNA can also be dissociated [63], [64], with State FS3 transiting to State FS5. For the ribosome in the non-canonical rotated conformation, the probabilities of the aminoacyl-tRNA dissociation in the two steps could be much larger than those for the ribosome in the canonical non-rotated conformation due partially to the fact that in the non-canonical rotated state the process of the codon recognition and that of the accommodation could be elongated greatly. Thus, multiple bindings of the ternary complex can occur in the stalled rotated state, which is consistent with the single-molecule data [29]. Since in State FS5 the 30S subunit is in the closed conformation, the ternary complex cannot bind efficiently, but EF-G.GTP binds efficiently. As the 30S subunit is now in the closed conformation, the GTP hydrolysis can induce efficiently the ribosomal unlocking (State FS6), inducing the 30S subunit transiting to open conformation and facilitating the reverse intersubunit rotation and Pi release. Due to the specific affinity of the 50S P site for the peptidyl-tRNA and the resistance of the mRNA duplex to the downstream movement of the 30S subunit, the reverse intersubunit rotation would cause the rotated State FS6 transiting to non-rotated State FS7, where the mRNA channel becomes tight. Then, the transitions from State FS7 to State C0 (indicated by the green arrow) are the same as those (inside the region bounded by broken lines) in Fig. 1.

If the accommodation is completed occasionally without aminoacyl-tRNA dissociation (State FS4), since the ribosome is not in the canonical non-rotated state the peptidyl transfer is inefficient, and since the 30S A site is occupied only EF-G.GTP can bind to State FS4. With the presence of the tRNA anticodon stem-loop bound to the 30S A site, i.e., with the 30S subunit in the closed conformation, after EF-G.GTP binding and then GTP hydrolysis (State FS8), the ribosomal unlocking occurs efficiently (State FS9), facilitating the reverse intersubunit rotation and Pi release. Due to the specific affinity of the 50S P and A sites for the peptidyl-tRNA and aminoacyl-tRNA, respectively, and the resistance of the mRNA duplex to the downstream movement of the 30S subunit, the reverse intersubunit rotation causes the rotated State FS9 to non-rotated State FS10. In State FS10, the mRNA channel becomes tight and the peptidyl-tRNA in the canonical P/P conformation induces the ribosome to be non-labile. After EF-G.GDP release and the efficient peptidyl transfer in the canonical non-rotated state, the ribosomal complex returns to Sate C0. Note that the transition from State FS3 to State C0 via State FS4, State FS8, State FS9 and State FS10 gives overlap of a tRNA pulse with the reverse rotation of the long pause, as observed experimentally (see Extended data Fig. 10c in Chen et al. [29], case 2). By contrast, the transition from State FS3 to State C0 via State FS5, State FS6 and State FS7 gives correlation of tRNA arrival and reverse rotation of the ribosome after the long rotated-state pause, as observed experimentally (see Extended data Fig. 10c in Chen et al. [29], case 1).

From State NF2, we have the similar pathway of state transitions to that shown in Fig. 4, but with the peptidyl-tRNA being paired with codon XXY. The peculiarity of the pathway (Fig. 4) is the presence of the long-paused rotated conformation of the ribosome, as observed by Chen et al. [29].

### 2.4 The comparison between the three models

The difference between the three models (Fig. 1, Fig. 2, Fig. 3) is in the transition of State H2 caused by the reverse intersubunit rotation. In the model of translation through the single-stranded mRNA, the reverse intersubunit rotation results in the transition of State H2 to State POST (with a probability $P_{E}^{\left(1\right)}$=1) (Fig. 1). The transition corresponds to the movement of the 30 S subunit relative to the tRNA-mRNA complex while the 50 S subunit being kept fixed to the two tRNAs.

In the model of translation through the duplex region of mRNA at the non-slippery site, the reverse intersubunit rotation results in the transition of State H2 also to State FC (with a probability $P_{F}^{\left(1\right)}$) besides to State POST (with a probability $P_{E}^{\left(1\right)}$, $P_{F}^{\left(1\right)}+P_{E}^{\left(1\right)}=1$) (Fig. 2). As mentioned just above, the transition to State POST corresponds to the movement of the 30S subunit relative to the tRNA-mRNA complex while the 50S subunit being kept fixed to the two tRNAs. By contrast, the transition to State FC corresponds to the movement of the 50S subunit relative to the two tRNAs while the 30S subunit being kept fixed to the tRNA-mRNA complex.

In the model of translation through the duplex region of mRNA at the slippery site, the reverse intersubunit rotation results in the transition of State H2 also to State LP (with a probability $P_{LP}^{\left(1\right)}$) besides to State POST (with a probability $P_{E}^{\left(1\right)}$) and State FC (with a probability $P_{F}^{\left(1\right)}$, $P_{LP}^{\left(1\right)}+P_{F}^{\left(1\right)}+P_{E}^{\left(1\right)}=1$) (Fig. 3). As mentioned just above, the transition to State POST corresponds to the movement of the 30S subunit relative to the tRNA-mRNA complex while the 50S subunit being kept fixed to the two tRNAs, whereas the transition to State FC corresponds to the movement of the 50S subunit relative to the two tRNAs while the 30S subunit being kept fixed to the tRNA-mRNA complex. By contrast, the transition to State LP corresponds to the movements of both the 30S and 50S subunits relative to the mRNA.

The three models can be commonly described by model of Fig. 3, where model of Fig. 2 corresponds to $P_{LP}^{\left(1\right)}$=0 and model of Fig. 1 corresponds to $P_{LP}^{\left(1\right)}$=$P_{F}^{\left(1\right)}$=0 or $P_{E}^{\left(1\right)}$=1.

## 3 Results and discussion

**Fig. 5:**
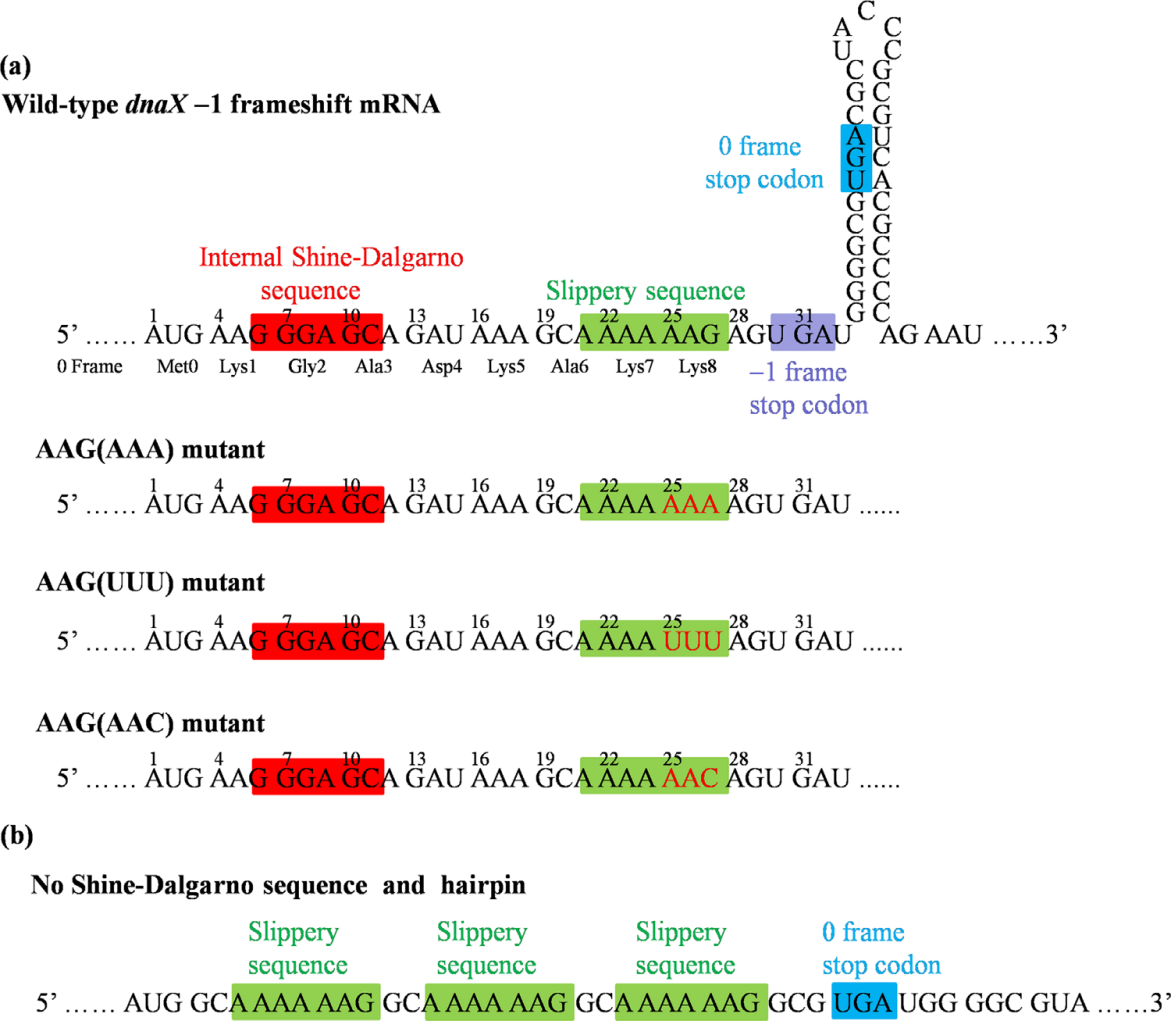
Some of the *dnaX* −1 frameshift mRNAs studied in this work. (a) WT and some mutant *dnaX* −1 frameshift mRNAs with both the internal Shine–Dalgarno sequence and hairpin. The hairpin regions of the mutant mRNAs are not shown. (b) The mutant mRNA with no internal Shine–Dalgarno sequence and hairpin.

In this work, we focus mainly on the explanation of the single-molecule experimental data of Chen et al. [29]. Thus, we focus our studies on the wild-type (WT) *dnaX* −1 frameshift mRNA and some mutant mRNAs used in the experiments, which contain both the internal SD sequence and hairpin, as shown in Fig. 5a. We also present predicted results with the mRNA in the absence of both SD sequence and hairpin, as shown in Fig. 5b.

### 3.1 Occurrence probability of the long-paused rotated state at the slippery site

Based on model of Fig. 3, at the slippery site (i.e., at codon AAA_24_, Fig. 5a) where both the SD-antiSD interaction and the downstream hairpin are present (see Fig. S2), a reverse intersubunit rotation can result in transition of State H2 to one of the three states, State POST, State FC and State LP. The transition to State POST requires overcoming the free energy change ($3E_{bp}$) of unwinding three mRNA base pairs and the free energy change ($\Delta E_{SD}^{\left(3\right)}$) of the SD-antiSD interaction induced by the movement of the ribosome from the position (with *d*=0) where the SD and antiSD have the largest interacting energy to the position (with *d*=3*p*, *p*=3.4 nm) where the SD and antiSD have nearly no interaction. The transition to State FC requires overcoming the binding energy ($E_{PE}^{\left(50S\right)}$) of the 50S E and P sites to the two tRNAs. The transition to State LP requires overcoming four free energy changes including the free energy change ($2E_{bp}$) of unwinding two mRNA base pairs, the free energy change ($\Delta E_{codon}$) of shifting the peptidyl-tRNA anticodon from pairing with codon XXY to pairing with codon XXX and breaking the interaction between deacylated tRNA anticodon and codon BCX, the free energy change (ΔtRNA) of bending the P-site peptidyl-tRNA in State LP, and the free energy change ($\Delta E_{SD}^{\left(2\right)}$) of the SD-antiSD interaction which is induced by the movement of the ribosome from the position with *d*=0 where the SD and antiSD have the largest interacting energy to the position with *d*=2*p* where the SD and antiSD have a smaller interacting energy, with $\Delta E_{SD}^{\left(2\right)}$<$\Delta E_{SD}^{\left(3\right)}$. The transition to State LP implies the occurrence of the long-paused rotated state. Thus, the occurrence probability of State LP caused by one reverse intersubunit rotation is calculated by

(1) $$P_{LP}^{\left(1\right)}=\frac{\exp\left[-\beta \left(\Delta E+\Delta E_{SD}^{\left(2\right)}+2E_{bp}\right)\right]}{\exp\left[-\beta \left(\Delta E+\Delta E_{SD}^{\left(2\right)}+2E_{bp}\right)\right]+\exp\left[-\beta \left(\Delta E_{SD}^{\left(3\right)}+3E_{bp}\right)\right]+\exp\left(-\beta E_{PE}^{\left(50S\right)}\right)},$$

where $\Delta E=\Delta E_{codon}+\Delta tRNA$ and $\beta^{-1}=k_{B}T$ is the thermal energy. Correspondingly, the occurrence probability of State POST, i.e., an effective translocation, caused by one reverse intersubunit rotation is calculated by

(2) $$P_{E}^{\left(1\right)}=\frac{\exp\left[-\beta \left(\Delta E_{SD}^{\left(3\right)}+3E_{bp}\right)\right]}{\exp\left[-\beta \left(\Delta E+\Delta E_{SD}^{\left(2\right)}+2E_{bp}\right)\right]+\exp\left[-\beta \left(\Delta E_{SD}^{\left(3\right)}+3E_{bp}\right)\right]+\exp\left(-\beta E_{PE}^{\left(50S\right)}\right)}$$

With Eqs. (1), (2) the occurrence probability of the long-paused rotated state during the translation is calculated by $P_{LP}=P_{LP}^{\left(1\right)}/\left(P_{LP}^{\left(1\right)}+P_{E}^{\left(1\right)}\right)$, which is rewritten as

(3) $$P_{LP}=\frac{\exp\left[-\beta \left(\Delta E-\Delta \Delta E_{SD}-E_{bp}\right)\right]}{\exp\left[-\beta \left(\Delta E-\Delta \Delta E_{SD}-E_{bp}\right)\right]+1},$$

where $\Delta \Delta E_{SD}=\Delta E_{SD}^{\left(3\right)}-\Delta E_{SD}^{\left(2\right)}$, and $\Delta E=\Delta E_{codon}+\Delta tRNA$ as defined in Eq. (1). Then, the occurrence probability of the normal translation at the slippery site (i.e., at codon AAA_24_) is calculated by $P_{Nor}=1-P_{LP}$.

Eq. (3) shows clearly that the occurrence probability of the long-paused rotated state is determined by the SD-antiSD interaction (characterized by $\Delta \Delta E_{SD}$) and the stability of the base pair of the downstream hairpin (characterized by *E*_*bp*_), as well as the codons at the slippery site (characterized by $\Delta E_{codon}$) and the bending of the P-site peptidyl-tRNA (characterized by ΔtRNA), while is independent of other factors such as the concentrations of EF-G.GTP and the ternary complex, which is consistent with the experimental data [29]. The single-molecule experimental data [29] showed that at the slippery site (i.e., at codon AAA_24_) of the WT mRNA, where both the SD-antiSD interaction and the downstream hairpin are present, the occurrence probability of the long-paused rotated state is 75%; for the mutant mRNA without the internal SD sequence, which corresponds to $\Delta \Delta E_{SD}$=0 in Eq. (3), the occurrence probability of the long-paused rotated state is 38%; and for the mutant mRNA without the downstream hairpin, which corresponds to *E*_*bp*_=0 in Eq. (3), the occurrence probability of the long-paused rotated state is 20%. With Eq. (3), by adjusting ΔE=3*k*_*B*_*T*, $\Delta \Delta E_{SD}$=1.6*k*_*B*_*T* and *E*_*bp*_=2.5*k*_*B*_*T* we obtain *P*_*LP*_=0.75 for the WT mRNA. Then, with ΔE=3*k*_*B*_*T*, $\Delta \Delta E_{SD}$=0 and *E*_*bp*_=2.5*k*_*B*_*T* we obtain *P*_*LP*_=0.38 for the mutant mRNA without the internal SD sequence; and with ΔE=3*k*_*B*_*T*, $\Delta \Delta E_{SD}$=1.6*k*_*B*_*T* and *E*_*bp*_=0 we obtain *P*_*LP*_=0.2 for the mutant mRNA without the downstream hairpin. The fitted value of *E*_*bp*_=2.5*k*_*B*_*T* implies that the free energy change of unwinding one mRNA base pair is about 1.5 kcal/mol, which is close to the value estimated in the literature [65].

From Eq. (3) it is clearly seen that the occurrence probability of the long-paused rotated state or the frequency of frameshifting increases with the increase of *E*_*bp*_, implying that increasing mRNA structure stability increases the frequency of frameshifting. Moreover, Eq. (3) shows that for the system with no SD sequence involvement, i.e., with $\Delta \Delta E_{SD}$=0, the efficiency *P*_*LP*_ is smaller than that with $\Delta \Delta E_{SD}$>0, explaining the experimental data showing that the frameshifting efficiency related to the system where only the IBV pseudoknot is present but no SD sequence is involved [24] is smaller than that for the system where both the stem loop and SD sequence are involved [29].

### 3.2 +2 translocation model versus +3 translocation model

Based on the experimental data, Chen et al. [29] proposed a model of the elongation pathway for translation at the slippery site (i.e., at codon AAA_24_) where both the SD-antiSD interaction and the downstream hairpin are present (see Fig. S1). The model argued that the translocation that is uncoupled with the reverse intersubunit rotation (called “uncoupled” translocation) can occur at codon AAA_24_ and the “uncoupled” translocation is the +3 translocation (i.e., the downstream translocation of the ribosome by 3 nucleotides along the mRNA). According to this model, for the AAG(AAA) mutant, i.e., changing AAG_27_ codon to AAA_27_ (Fig. 5a), after the “uncoupled” translocation the peptidyl-tRNA^Lys^(AAA_24_) codon pair is in the P site and the AAA_27_ codon is in the A site that is now available for aminoacyl-tRNA binding. Since whether after the −1 slippage or not the codon in the A site is always AAA, both the AAA_26_ codon (−1 frame) and AAA_27_ codon (0 frame) have the same interaction energy with the aminoacyl-tRNA^Lys^. Thus, it would be expected that in the pathway of the “uncoupled” translocation (Fig. S1), the probability of the translation in 0 frame should be larger than or at least equal to that of the translation in −1 frame. However, this is inconsistent with the experimental data [29], as discussed below. The experimental data [29] showed that for the WT mRNA about *F*_*UT*_=71–75% fraction of the elongating ribsosmes proceed in the pathway of the “uncoupled” translocation (with 71% of elongating ribosomes exhibiting>4 Cy5-tRNA^Lsy^ pulses or with the frameshifting percentage of about 75%) and for the AAG(AAA) mutant mRNA the –1 frameshifting percentage is about *F*_*FS*_=49%. Since for both the WT mRNA and AAG(AAA) mutant, the elongating ribosomes should have the same fraction (*F*_*UT*_=71–75%) that proceed in the pathway of the “uncoupled” translocation, it is thus expected that for the AAG(AAA) mutant the non-frameshifting percentage is about *F*_*NF*_=*F*_*UT*_−*F*_*FS*_=22–26% in the pathway of the “uncoupled” translocation. In other words, in the pathway of the “uncoupled” translocation, about 22–26% ribosomes that enter the stalled state do not frameshift, while the experimental data [29] showed that about 49% ribosomes that enter the stalled state frameshift, with the former being about 2-fold smaller than the latter. This is inconsistent with the deduction from the experimental data showing that for the AAG(AAA) mutant, in the pathway of the “uncoupled” translocation, the probability of the translation in 0 frame should be larger than or at least equal to that of the translation in −1 frame (see just above). The inconsistency thus argues against the +3 translocation proposal.

By contrast, the experimental data for the AAG(AAA) mutant supports the incomplete +2 translocation, which is consistent with our model (Fig. 4) predicting that the probability of the translation in −1 frame is larger than in 0 frame. For other mutant sequences such as the AAG(UUU) mutant (changing AAG_27_ to UUU_27_) and AAG(AAC) mutant (changing AAG_27_ to AAC_27_) with the codon for −1 frame in the A site being different from the codon for 0 frame (Fig. 5a), the two codons have different interaction energies with their corresponding cognate tRNAs, which plays an important role in determining the probability of the translation in 0 frame and that of the translation in −1 frame in the pathway of the +2 translocation (Fig. 4). This could provide an explanation of the experimental data in Chen et al. [29]. For the WT sequence, the AAA_26_ codon (−1 frame) has the larger interaction strength with the UUU anticodon of the aminoacyl-tRNA^Lys^ than the AAG_27_ codon (0 frame). Thus, the probability of the translation in −1 frame is expected to be much larger than that of the translation in 0 frame in the pathway of the +2 translocation. For the AAG(AAA) mutant, the same AAA codon for −1 frame and 0 frame give the same interaction energy with the UUU anticodon of the aminoacyl-tRNA^Lys^, inducing the probability of the translation in −1 frame to be smaller than that for the WT sequence. For the AAG(UUU) mutant, the further reduction of the probability of the translation in −1 frame relative to that for the AAG(AAA) mutant [29] could be due to the fact that the UUU_27_ codon (0 frame) has a stronger interaction than the AUU_26_ codon (−1 frame) with their corresponding cognate tRNAs.

The experimental data on tRNA sampling dynamics and slippage during frameshifting (Extended data Fig. 9 in Chen et al. [29]) are explained as follows. The available biochemical data showed that the binding rate of a near-cognate tRNA is similar to that of the cognate tRNA to the A site of the ribosome [63], [64]. Thus, for the AAG(UUU) mutant, it is expected that after the +2 translocation at codon AAA_24_, the arrival time of the near-cognate aminoacyl-tRNA^Phe^ to the A-site AUU_26_ codon (−1 frame) would be similar to that of the cognate aminoacyl-tRNA^Lys^ to the A-site AAA_26_ codon (−1 frame) for the WT sequence. Consequently, after the +2 translocation the tRNA^Phe^ arrival time for the AAG(UUU) mutant could be similar to tRNA^Lys^ arrival time for the WT sequence. Moreover, for the AAG(UUU) mutant, after the binding of tRNA^Phe^ to the A-site AUU_26_ codon (−1 frame) the stronger interaction of the AAA anticodon of tRNA^Phe^ with the UUU codon induces slippage of the two tRNAs to the 0 frames. Consequently, after the +2 translocation at codon AAA_24_ the mean lifetime of tRNA^Phe^ sampling for the AAG(UUU) mutant could be also similar to that of tRNA^Lys^ sampling for the WT sequence. In addition, for Cy5-tRNA^Lys^ transit through the AAG(UUU) mutant, because both the sampling of cognate tRNA^Ile^ to the A-site AUU_26_ codon (−1 frame) and the sampling of cognate tRNA^Phe^ to the A-site UUU_27_ codon (0 frame) are invisible, only three Lys pulses can be observed, consistent with the experimental data [29]. For Cy5-tRNA^Lys^ transit through the AAG(AAC) mutant, after the +2 translocation at codon AAA_24_, because the binding rate of the near-cognate tRNA^Asn^ to the A-site AAA_26_ codon (−1 frame) is similar to the cognate tRNA^Lys^ to the A-site AAA_26_ codon, about half fraction of Cy5-tRNA^Lys^ samplings to the A-site AAA_26_ codon can occur. On the other hand, the +2 translocation contains about 71% fraction of the total translocations [29]. Thus, it is estimated that the probability of observing more than three Lys pulses is about 35%, which is slightly larger than the experimental data of about 25% [29]. Assuming that the Cy5-labeling may reduce the tRNA binding rate, it is expected that the probability of observing more than three Lys pulses is slightly smaller than 35% estimated above.

It should be mentioned that based on the fact that the binding rate of the near-cognate tRNA is similar to that of the cognate tRNA to the A site of the ribosome [63], [64], it is expected that both the +2 translocation model and the +3 translocation model would give the similar results on the tRNA sampling dynamics for the mRNAs such as AAG(UUU) mutant and AAG(AAC) mutant used in Extended data Fig. 9 of Chen et al. [29]. Thus, based only on the experimental data given in Extended data Fig. 9 of Chen et al. [29], we cannot determine which of the two models is more reasonable. However, as discussed above, the experimental data for the AAG(AAA) mutant, which gives the same A-site codon for both −1 frame and 0 frame, are inconsistent with the +3 translocation model but are consistent with the +2 translocation model.

### 3.3 tRNA transit and sampling dynamics in the long-paused rotated state

#### 3.3.1 Mean number of aminoacyl-tRNA^Lys^ samplings to the long-paused rotated state

With the similar procedure to that used before [66], we easily derive that the distribution of times for transition from State FS2 to State FS3 (Fig. 4) has the form, $f_{2\to 3}(t)=k_{20}\exp\left(-k_{20}t\right)$, and the distribution of times of the aminoacyl-tRNA dissociation during transition from State FS2 to State FS3 has the form, $g_{2\to 3}(t)=k_{21}\exp\left(-k_{21}t\right)$. With $f_{2\to 3}(t)$ and $g_{2\to 3}(t)$, the probability of the aminoacyl-tRNA dissociation during transition from State FS2 to State FS3 is calculated by $P_{2\to 3}=\int_{0}^{\infty }dtg_{2\to 3}(t)\int_{t}^{\infty }dt'f_{2\to 3}(t')$, which is rewritten as

(4) $$P_{2\to 3}=\frac{k_{21}}{k_{21}+k_{20}}$$

With the dissociation probability $P_{2\to 3}$, the mean number of the aminoacyl-tRNA samplings to the long-paused rotated state in Fig. 4 is calculated by $N=1+P_{2\to 3}+{\left(P_{2\to 3}\right)}^{2}+\cdot \cdot \cdot =1/\left(1-P_{2\to 3}\right)$. With Eq. (4), *N* has the form

(5) $$N=\frac{k_{20}+k_{21}}{k_{20}}$$

From Eq. (5) it is seen that the mean number *N* is only determined by two rate constants *k*_20_ and *k*_21_, which are independent of the concentrations of EF-G.GTP and the ternary complex (see Fig. 4). Thus, *N* remains constant at the various factor concentrations, which is consistent with the single-molecule experimental data (Extended Data Fig. 8c in Chen et al. [29]). From the measured value of *N≈*2 [29], with Eq. (5) we obtain $k_{21}/k_{20}$≈1.

#### 3.3.2 Mean lifetime of aminoacyl-tRNA^Lys^ bound to the long-paused rotated state

Then we derive equation of the mean lifetime of the aminoacyl-tRNA bound to the long-paused rotated state in Fig. 4. We easily derive that the distribution of times for transition from State FS3 to State FS4 has the form, $f_{3\to 4}(t)=k_{22}\;\exp\left(-k_{22}t\right)$, and the distribution of times of the aminoacyl-tRNA dissociation during transition from State FS3 to State FS4 has the form, $g_{3\to 4}(t)=k_{23}\;\exp\left(-k_{23}t\right)$. With $f_{3\to 4}(t)$ and $g_{3\to 4}(t)$, we obtain that the probability of the aminoacyl-tRNA dissociation during transition from State FS3 to State FS4 has the form

(6) $$P_{3\to 4}=\frac{k_{23}}{k_{23}+k_{22}}$$

For the case that the aminoacyl-tRNA is dissociated during the transition from State FS2 to State FS3, the mean lifetime of the aminoacyl-tRNA bound to the long-paused rotated state is calculated by $T_{1}=\int_{0}^{\infty }dtf_{2\to 3}(t)\int_{0}^{t}dt't'g_{2\to 3}(t')$, which is rewritten as

(7) $$T_{1}=\frac{1}{k_{21}}-\frac{k_{20}k_{20}+2k_{20}k_{21}}{k_{21}{\left(k_{21}+k_{20}\right)}^{2}}$$

For the case that the aminoacyl-tRNA is dissociated during the transition from State FS3 to State FS4, the mean lifetime of the aminoacyl-tRNA bound to the long-paused rotated state is calculated by $T_{2}=1/k_{20}+\int_{0}^{\infty }dtf_{3\to 4}(t)\int_{0}^{t}dt't'g_{3\to 4}(t')$, which is rewritten as

(8) $$T_{2}=\frac{1}{k_{20}}+\frac{1}{k_{23}}-\frac{k_{22}k_{22}+2k_{22}k_{23}}{k_{22}{\left(k_{23}+k_{22}\right)}^{2}}$$

For the case that the aminoacyl-tRNA is not dissociated during the transition from State FS3 to State FS4, the mean lifetime of the aminoacyl-tRNA bound to the long-paused rotated state is calculated by

(9) $$T_{3}=\frac{1}{k_{20}}+\frac{1}{k_{22}}+\frac{1}{k_{26}}+\frac{1}{k_{27}}+\frac{1}{k_{28}}$$

Then, the mean lifetime of the aminoacyl-tRNA bound to the long-paused rotated state in Fig. 4 is calculated by

(10) $$T_{tRNA}=\frac{\left(N-1\right)T_{1}+P_{3\to 4}T_{2}+\left(1-P_{3\to 4}\right)T_{3}}{N}$$

With Eqs. (5), (6), (7), (8), (9), Eq. (10) can be rewritten as

(11) $$\begin{array}{c} T_{tRNA}=\frac{1}{k_{20}+k_{21}}-\frac{k_{20}\left(k_{20}+2k_{21}\right)}{{\left(k_{21}+k_{20}\right)}^{3}}\\ \;+\frac{k_{20}k_{23}}{\left(k_{20}+k_{21}\right)\left(k_{23}+k_{22}\right)}\left(\frac{1}{k_{20}}+\frac{1}{k_{23}}-\frac{k_{22}k_{22}+2k_{22}k_{23}}{k_{22}{\left(k_{23}+k_{22}\right)}^{2}}\right)\\ \;+\frac{k_{20}k_{22}}{\left(k_{20}+k_{21}\right)\left(k_{23}+k_{22}\right)}\left(\frac{1}{k_{20}}+\frac{1}{k_{22}}+\frac{1}{k_{26}}+\frac{1}{k_{27}}+\frac{1}{k_{28}}\right) \end{array}$$

The experimental data showed that the transitions are mainly along the pathway of transition from State FS3 to State FS5, implying that $P_{3\to 4}$ approaches nearly 1. Thus, from Eq. (6) we note that *k*_23_»*k*_22_. This implies that the third term in the right-hand side of Eq. (11) is much smaller than the other two terms. Moreover, rate constants *k*_20_, *k*_21_, *k*_22_ and *k*_23_ are independent of the concentrations of EF-G.GTP and the ternary complex (see Fig. 4). Thus, Eq. (11) implies that the mean lifetime *T*_*tRNA*_ remains constant at the various factor concentrations, which is also consistent with the single-molecule experimental data (Extended Data Fig. 8c in Chen et al. [29]).

#### 3.3.3 Mean arrival time of aminoacyl-tRNA^Lys^ to the long-paused rotated state

In State FS1 with open 30S subunit (Fig. 4), EF-G and the ternary complex compete for the binding to the ribosome. Denoting by $k_{b1}^{\left(G\right)}$ the binding rate of EF-G.GTP to the long-paused rotated state, [EF-G] the concentration of EF-G.GTP, $k_{b1}^{\left(TC\right)}$ the binding rate of the ternary complex cognate to codon AAA to the long-paused rotated state of the ribosome with open 30S subunit and [TC] the concentration of the ternary complex cognate to codon AAA, the probability of EF-G.GTP binding can be calculated by

(12) $$P^{\left(G\right)}=\frac{k_{b1}^{\left(G\right)}[\text{EF}-G]}{k_{b1}^{\left(G\right)}[\text{EF}-G]+k_{b1}^{\left(TC\right)}[\mathrm{TC}]}$$

Since in the solution except tRNA^Lys^ there is no other tRNA that is cognate or near-cognate to codon AAA [29], we have neglected the binding of other tRNAs except tRNA^Lys^ to codon AAA_26_ in Eq. (12). With probability *P*^(*G*)^, the mean number of EF-G.GTP molecules that can bind to the long-paused rotated state before tRNA^Lys^ binding can be calculated by $N^{\left(G\right)}=P^{\left(G\right)}+{\left(P^{\left(G\right)}\right)}^{2}+\cdot \cdot \cdot$, which is rewritten as

(13) $$N^{\left(G\right)}=\frac{P^{\left(G\right)}}{1-P^{\left(G\right)}}$$

With *N*^(*G*)^ EF-G.GTP molecules that can bind to the long-paused rotated state before tRNA^Lys^ binding, the mean arrival time of tRNA^Lys^ to the long-paused rotated state can be calculated by

(14) $$\tau =N^{\left(G\right)}T^{\left(G\right)}+\frac{1}{k_{b1}^{\left(TC\right)}[\mathrm{TC}]},$$

where *T*^(*G*)^ is the mean EF-G lifetime bound to the long-paused rotated state. With Eqs. (12), (13), Eq. (14) is rewritten as

(15) $$\tau =\frac{1+T^{\left(G\right)}k_{b1}^{\left(G\right)}[\text{EF}-G]}{k_{b1}^{\left(TC\right)}[\mathrm{TC}]},$$

where we show explicitly the dependence of τ on [EF-G] and [TC], while the other parameters are independent of [EF-G] and/or [TC].

**Fig. 6:**
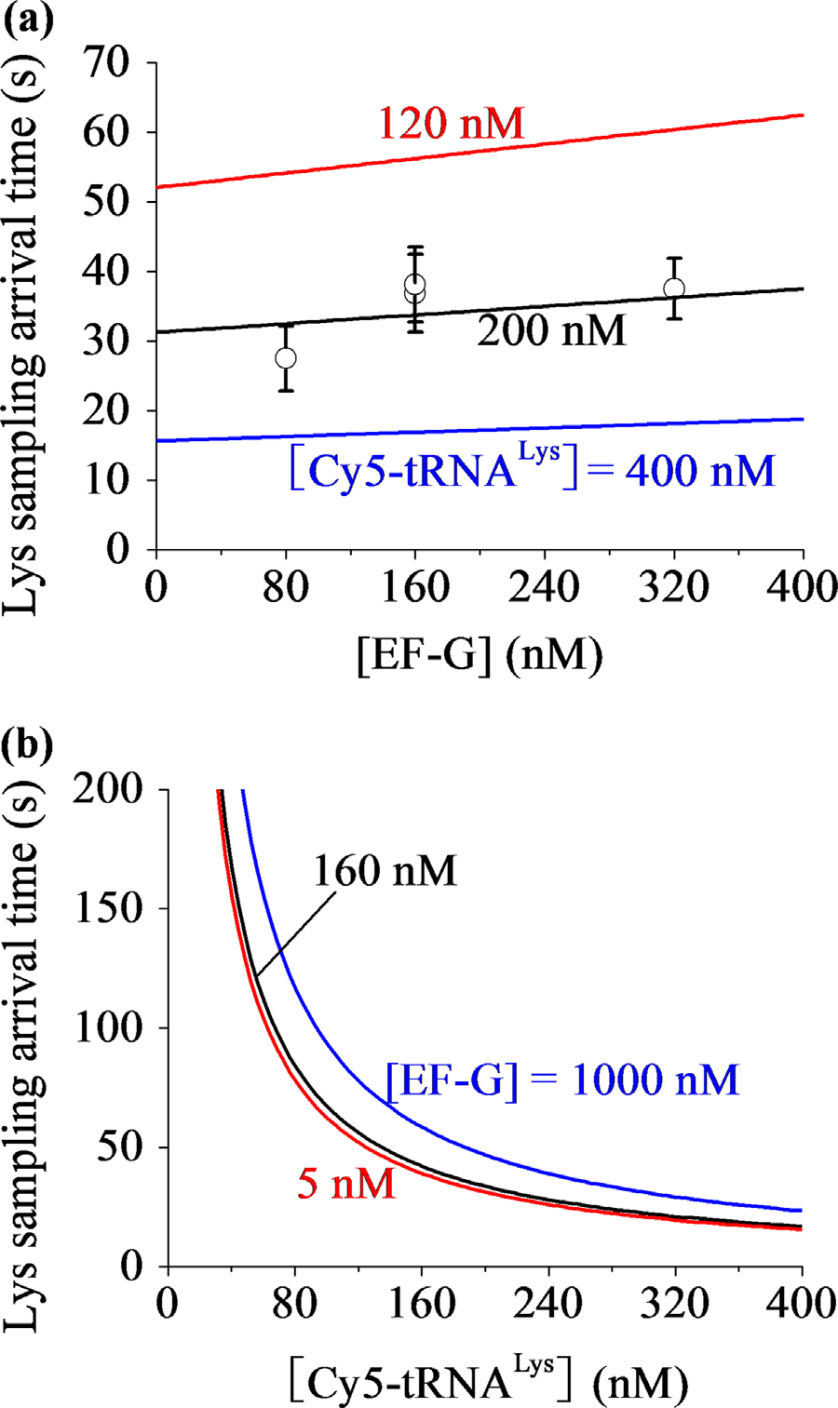
Mean arrival time of the cognate ternary complex to the long-paused rotated state. (a) The mean arrival time versus [EF-G] at different concentrations of the cognate ternary complex. The dots are taken from the experimental data (Extended data Fig. 8c in Chen et al. [29]). (b) The mean arrival time versus the concentration of the cognate ternary complex at different concentrations of EF-G.

With Eq. (15), by adjusting $T^{\left(G\right)}k_{b1}^{\left(G\right)}$=0.5 ${\mu M}^{-1}$ and $k_{b1}^{\left(TC\right)}$=0.16 ${\mu M}^{-1}s^{-1}$ we obtain that the results of the mean arrival time of tRNA^Lys^ to the long-paused rotated state, τ, versus [EF-G] at the concentration of tRNA^Lys^, [TC]=200 nM, are consistent with the experimental data (Extended data Fig. 8c in Chen et al. [29]) (Fig. 6a). In Fig. 6a we also show the predicted results of τ versus [EF-G] at other values of [TC]. In Fig. 6b we show the predicted results of τ versus [TC] for different values of [EF-G].

### 3.4 EF-G sampling dynamics

#### 3.4.1 During translation at non-slippery sites and without the SD-antiSD interaction

Based on our model, for the case of the translation at non-slippery sites and with no SD-antiSD interaction, the probability of effective translocation, $P_{E}^{\left(1\right)}$≈1 (see Section S2), giving the number of EF-G bindings per codon, $N_{1}^{\left(G\right)}$≈1. This is consistent with the single-molecule experimental data (about 1–1.5) at codons except near codon AAA_24_ (see Fig. 3b in Chen et al. [29]). Since after EF-G.GTP binding GTP hydrolysis is fast and the ribosomal unlocking facilitates rapid reverse ribosomal rotation [43], the mean EF-G lifetime can be approximately calculated by (see Fig. 1)

(16) $$T_{1}^{\left(G\right)}=\frac{1}{k_{4}}+\frac{1}{k_{6}}+\frac{1}{k_{7}}$$

From the available biochemical data, we have *k*_4_=35 $s^{-1}$, *k*_6_=5 $s^{-1}$ and *k*_7_=20 $s^{-1}$ [43], [67]. With these values, from Eq. (16) we obtain $T_{1}^{\left(G\right)}$=0.28 s, which is close to the experimental data of about 0.2 s (see Fig. 3b in Chen et al. [29]).

#### 3.4.2 During translation at the non-slippery site and with both the SD-antiSD interaction and hairpin

For the case of the translation at the non-slippery site where both the SD-antiSD interaction and the downstream hairpin are present, i.e., at codon AAG_24_ for A21G-A24G mutant (refer to Fig. S2), the probability $P_{E}^{\left(1\right)}$ becomes smaller than 1, giving the number of EF-G bindings per codon larger than 1 (see Fig. 2). The mean number of futile translocations is then calculated by $N_{F}=\left(1-P_{E}^{\left(1\right)}\right)+{\left(1-P_{E}^{\left(1\right)}\right)}^{2}+\cdot \cdot \cdot$=$\left(1-P_{E}^{\left(1\right)}\right)/P_{E}^{\left(1\right)}$. Thus, the mean number of EF-G bindings per codon is calculated by $N_{2}^{\left(G\right)}=1+N_{F}$, which is rewritten as

(17) $$N_{2}^{\left(G\right)}=\frac{1}{P_{E}^{\left(1\right)}}$$

Since the rate constant *k*_5_ of the ribosomal unlocking is large (about 35 $s^{-1}$ [43], [67] ), the mean EF-G lifetime (see Fig. 2) can thus be approximately calculated by

(18) $$T_{2}^{\left(G\right)}=\frac{1}{N_{2}^{\left(G\right)}}\left[\left(N_{2}^{\left(G\right)}-1\right)\left(\frac{1}{k_{14}}+\frac{1}{k_{r}}\right)+T_{1}^{\left(G\right)}\right],$$

where *k*_*r*_ is the rate of EF-G.GDP releasing from hybrid State FH.

From the single-molecule experimental data, we have $N_{2}^{\left(G\right)}$=4 at codon AAG_24_ for A21G-A24G mutant (see Fig. 3c in Chen et al. [29]). From Eq. (17) we obtain $P_{E}^{\left(1\right)}$=0.25, implying that the SD-antiSD interaction and the unwinding of the downstream hairpin induces the effective-translocation probability $P_{E}^{\left(1\right)}$ at codon AAG_24_ to be about 0.25. Previous single-molecule data showed that the release of EF-G.GDP from the pretranslocation state is slower than that from the posttrasnlocation state [50], giving $1/k_{14}+1/k_{r}$>$T_{1}^{\left(G\right)}$. Thus, from Eq. (18) it is expected that $T_{2}^{\left(G\right)}$>$T_{1}^{\left(G\right)}$, which is consistent with the experimental data showing that the mean EF-G lifetime at codon AAG_24_, where the SD-antiSD interaction is present, is about 1.3-fold – 2-fold larger than those at other codons, where no SD-antiSD interaction is present (see Fig. 3c in Chen et al. [29]).

#### 3.4.3 During translation at the slippery site and with both the SD-antiSD interaction and hairpin

For the case of the translation at the slippery site where both the SD-antiSD interaction and the downstream hairpin are present, i.e., at codon AAA_24_ for the WT (refer to Fig. S2), the mean number of EF-G bindings in an elongation cycle (see Fig. 3, Fig. 4) can be calculated by

(19) $$N_{3}^{\left(G\right)}=N_{2}^{\left(G\right)}+P_{LP}N_{LP}^{\left(G\right)},$$

where $N_{2}^{\left(G\right)}$ is calculated by Eq. (17) but with $P_{E}^{\left(1\right)}$ being replaced by $P_{E}^{\left(1\right)}+P_{LP}^{\left(1\right)}$ and $N_{LP}^{\left(G\right)}$ is the mean number of EF-G molecules that can bind to the long-paused rotated state (State FS1, Fig. 4) in an elongation cycle. Correspondingly, the mean EF-G lifetime at the slippery site can be approximately calculated by

(20) $$T_{3}^{\left(G\right)}=\frac{N_{2}^{\left(G\right)}T_{2}^{\left(G\right)}+P_{LP}N_{LP}^{\left(G\right)}T^{\left(G\right)}}{N_{2}^{\left(G\right)}+P_{LP}N_{LP}^{\left(G\right)}},$$

where $N_{2}^{\left(G\right)}$ and $T_{2}^{\left(G\right)}$ are calculated by Eqs. (17), (18), respectively, but with $P_{E}^{\left(1\right)}$ being replaced by $P_{E}^{\left(1\right)}+P_{LP}^{\left(1\right)}$, and *T*^(*G*)^ is defined in Eq. (14).

As determined just above, at the non-slippery site and with both the SD-antiSD interaction and downstream hairpin, the probability of effective translocation caused by one reverse intersubunit rotation is about 0.25. Thus, for an approximation, at the slippery site $P_{E}^{\left(1\right)}$ can be calculated by $P_{E}^{\left(1\right)}=0.25\left(1-P_{LP}^{\left(1\right)}\right)$. Then, $P_{LP}^{\left(1\right)}$ can be calculated from $P_{LP}=P_{LP}^{\left(1\right)}/\left(P_{LP}^{\left(1\right)}+P_{E}^{\left(1\right)}\right)$=$P_{LP}^{\left(1\right)}/\left[P_{LP}^{\left(1\right)}+0.25\left(1-P_{LP}^{\left(1\right)}\right)\right]$. With *P*_*LP*_=0.75 [29], we obtain $P_{LP}^{\left(1\right)}$=0.43 and $P_{E}^{\left(1\right)}$=0.14. From Eq. (17) but with $P_{E}^{\left(1\right)}$ being replaced by $P_{E}^{\left(1\right)}+P_{LP}^{\left(1\right)}$, we obtain $N_{2}^{\left(G\right)}$=1.75. With this value of $N_{2}^{\left(G\right)}$, $N_{3}^{\left(G\right)}$=5.3 (see Fig. 3b in Chen et al. [29]) and *P*_*LP*_=0.75 [29], from Eq. (19) we have the mean number of EF-G bindings in an elongation cycle under the condition of Fig. 3b in Chen et al. [29], $N_{LP}^{\left(G\right)}$=4.73.

With $N_{LP}^{\left(G\right)}$=4.73, $N_{2}^{\left(G\right)}$=1.75, $T_{2}^{\left(G\right)}$≈0.25 s (see Fig. 3c in Chen et al. [29]), $T_{3}^{\left(G\right)}$≈0.9 s (see Fig. 3b in Chen et al. [29]) and *P*_*LP*_=0.75 [29], from Eq. (20) we obtain the mean lifetime of EF-G bound to the long-paused rotated state, *T*^(*G*)^≈1.22 s. With *T*^(*G*)^≈1.22 s, from $T^{\left(G\right)}k_{b1}^{\left(G\right)}$=0.5 ${\mu M}^{-1}$ (see Section 3.3.3) we obtain $k_{b1}^{\left(G\right)}$≈0.41 ${\mu M}^{-1}s^{-1}$, which is about 4-fold smaller than the binding rate to the canonical rotated state (about 1.56 ${\mu M}^{-1}s^{-1}$, see below).

In addition, it is important to see from Fig. 4 that both the reverse intersubunit rotation in the transition from State FS6 to State FS7 and that in the transition from State FS9 to State FS10 are catalyzed by EF-G.GTP binding and then GTP hydrolysis, implying that the long-paused non-canonical rotated state is finally resolved by EF-G, which is consistent with the single-molecule experimental data [29].

### 3.5 Mean rotated-state lifetimes

It was well characterized that after the peptidyl transfer and before EF-G.GTP binding, the pretranslocation ribosomal complex can transit spontaneously between the non-rotated and rotated states, with the two states being in thermodynamic equilibrium and the majority being in the rotated states [37], [38], [39]. In other words, at low concentration of EF-G.GTP (≤480 nM) as used in the experiments of Chen et al. [29], after transition to the rotated state the ribosome can occasionally transit backward to the non-rotated state and then rapidly transit forward to the rotated state again before the slow binding of EF-G.GTP. However, it was observed in the experiments that after transition to the rotated state the ribosome is kept in the rotated state in the long period before EF-G.GTP binding [29], implying that the short-lived non-rotated state that occurs after the occasional backward transition from the rotated state cannot be resolved from the FRET signal in the experiments [29]. Thus, in the analysis in this work focusing on the explanation of the experimental data of Chen et al. [29] we neglect the backward transition from State H0 to State C0 (see Fig. 1). In addition, previous single-molecule experimental data of the same group [50] showed that the binding rate of EF-G.GTP to the non-rotated state is about 0.84 ${\mu M}^{-1}s^{-1}$, giving the rate constant *k*_1_<0.4 $s^{-1}$ at [EF-G] ≤480 nM (see Fig. 1). By contrast, the smFRET data of the same group [68] showed the rate constant *k*_01_>2 $s^{-1}$. Thus, in the analysis in this work we neglect the transition from State C0 to State C and only consider the transition from State C0 to State H0.

After the futile translocation (see Fig. 2, Fig. 3) or incomplete translocation (see Fig. 3), the ribosome is in the non-rotated state (State FC or State LP) and the lifetimes of the non-rotated State FC and State LP are $1/k_{14}$ and $1/k_{16}$, respectively. Since EF-G.GDP facilitates the transition of the non-rotated state to rotated state and then stabilizes the rotated state [40], [41], it is thus expected that the rate constants *k*_14_ and *k*_16_ are larger than *k*_01_, implying that the lifetimes of State FC and State LP are shorter than State C0. As mentioned above, before EF-G.GTP binding State H0 can occasionally transit backward to State C0, but the short lifetime of State C0 cannot be resolved from the FRET signal in the experiments of Chen et al. [29]. Thus, it is expected that the lifetimes of State FC and State LP also cannot be resolved in the experiments [29]. Under this consideration, the lifetimes of the rotated state for different cases are studied as follows.

#### 3.5.1 During translation at non-slippery sites and without the SD-antiSD interaction

For the case of the translation at the non-slippery site and with no SD-antiSD interaction, the probability of effective translocation, $P_{E}^{\left(1\right)}$≈1 (see Section S2), with the elongation pathway being shown in Fig. 1. The mean rotated-state lifetime is calculated by $T_{R1}=1/k_{2}+1/k_{4}+1/k_{5}$. As after EF-G.GTP binding, GTP hydrolysis is fast [43], the rate constant *k*_2_ is approximately determined only by the binding of EF-G.GTP, with $k_{2}=k_{b}^{\left(G\right)}[\text{EF}-G]$, where $k_{b}^{\left(G\right)}$ is the binding rate of EF-G.GTP to State H0 and [EF-G] the concentration of EF-G.GTP. In addition, the available biochemical data showed that after GTP hydrolysis by EF-G bound to the rotated state, the ribosomal unlocking occurs rapidly, with a rate of about 35 $s^{-1}$ [43], [67] that is much larger than the binding rate of EF-G.GTP at the concentrations used in the experiment [29]. Moreover, after the ribosomal unlocking the reverse intersubunit rotation is fast [43]. Thus, for approximation, the mean rotated-state lifetime can be calculated by

(21) $$T_{R1}=\frac{1}{k_{b}^{\left(G\right)}[\text{EF}-G]}$$

It is clearly seen from Eq. (21) that for the translation of the codons that are not at the slippery site and with no SD-antiSD interaction, the mean rotated-state lifetime is inversely proportional to [EF-G] and independent of the concentration of the ternary complex, which is consistent with the single-molecule experimental data (see Extended Data Fig. 5d and e in Chen et al. [29]). From the experimental data (see Extended Data Figs. 4 and 5 in Chen et al. [29]), we have *T*_*R*1_≈8 s at [EF-G]=80 nM. Then, with Eq. (21) we obtain the binding rate to the canonical rotated state, $k_{b}^{\left(G\right)}$=1.56 ${\mu M}^{-1}s^{-1}$, which is close to the value (about 1.9 ${\mu M}^{-1}s^{-1}$) determined in another paper of Chen et al. [50] and is about 4-fold larger than the binding rate to the non-canonical rotated state ($k_{b1}^{\left(G\right)}$=0.41 ${\mu M}^{-1}s^{-1}$, see Section 3.4.3).

#### 3.5.2 During translation at the non-slippery site and with both the SD-antiSD interaction and hairpin

For the case of the translation at the non-slippery site with both the SD-antiSD interaction and the downstream hairpin, i.e., at codon AAG_24_ for A21G-A24G mutant, the probability of effective translocation, $P_{E}^{\left(1\right)}$, becomes smaller than 1, with the elongation pathway being shown in Fig. 2. Then, the mean rotated-state lifetime can be approximately calculated by $T_{R2}=T_{R1}+\left(1-P_{E}^{\left(1\right)}\right)/k_{15}+{\left(1-P_{E}^{\left(1\right)}\right)}^{2}/k_{15}+\cdot \cdot \cdot$. The rate constant *k*_15_ can be calculated by the relation, $1/k_{15}=1/\left(k_{b}^{\left(G\right)}[\text{EF}-G]\right)+1/k_{r}$, where *k*_*r*_ is the rate of EF-G.GDP releasing from State FH. Thus, the mean rotated-state lifetime *T*_*R*2_ approximately has the form

(22) $$T_{R2}=T_{R1}+\frac{\left(1-P_{E}^{\left(1\right)}\right)}{P_{E}^{\left(1\right)}}\left(\frac{1}{k_{b}^{\left(G\right)}[\text{EF}-G]}+\frac{1}{k_{r}}\right)$$

With Eqs. (21 and 22) is rewritten as

(23) $$T_{R2}=\frac{1}{P_{E}^{\left(1\right)}k_{b}^{\left(G\right)}[\text{EF}-G]}+\frac{1-P_{E}^{\left(1\right)}}{P_{E}^{\left(1\right)}k_{r}}$$

As determined above, $P_{E}^{\left(1\right)}$=0.25 (see Section 3.4.2). Thus, from Eq. (23) the mean rotated-state lifetime *T*_*R*2_ at the non-slippery site where both the SD-antiSD interaction and downstream hairpin are present is more than 4-fold larger than that without the SD-antiSD interaction, which is consistent with the single-molecule experimental data of about 4-fold – 5-fold (see Extended Data Fig. 6c in Chen et al. [29]).

In addition, based on Fig. 2 it is noted that the mean time of the departure of deacylated Cy3-tRNA^Val^ for GCA_21_ (Ala) to GUA_21_ (Val) mutant relative to the arrival of Cy5-tRNA^Lys^ at codon Lys7 can be approximately calculated by $T_{de}=T_{NR}+T_{R2}$, where *T*_*NR*_ is the mean lifetime of the non-rotated state. From the experimental data (see Fig. 1d or Extended Data Figs. 4 and 5 in Chen et al. [29]) *T*_*NR*_≈12 s at 1 μM ternary complex and *T*_*R*1_≈8 s at [EF-G]=80 nM. From Eq. (21) we have ${\left(k_{b}^{\left(G\right)}[\text{EF}-G]\right)}^{-1}$=*T*_*R*1_≈8 s. With above values of *T*_*NR*_ and $k_{b}^{\left(G\right)}[\text{EF}-G]$, from Eq. (23) and $T_{de}=T_{NR}+T_{R2}$ we obtain *T*_*de*_≥44 s, which is in quantitative agreement with the experimental data of 45±11 s (see Fig. 2a in Chen et al. [29]). Moreover, from Eq. (23) it is seen that as [EF-G] increases the mean rotated-state lifetime *T*_*R*2_ decreases, implying that *T*_*de*_ decreases with increasing [EF-G], which is also consistent with the experimental data (see Fig. 2a in Chen et al. [29]).

#### 3.5.3 During translation at the slippery site and with both the SD-antiSD interaction and hairpin

For the case of the translation at the slippery site (i.e., at codon AAA_24_) and with both the SD-antiSD interaction and downstream hairpin, the elongation pathway is shown in Fig. 3, Fig. 4. For the transitions inside the box of Fig. 3, the mean lifetime of the rotated state can still be calculated by Eq. (23) but with $P_{E}^{\left(1\right)}$ being replaced by $P_{E}^{\left(1\right)}+P_{LP}^{\left(1\right)}$. During the transition from State FS through State FS3, the mean lifetime of the rotated state is calculated by $N\left(\tau +T_{tRNA}\right)$, where *N* is the mean number of the aminoacyl-tRNA samplings to the long-paused rotated state in Fig. 4, which is calculated by Eq. (5), τ is the mean arrival time of tRNA^Lys^ to the long-paused rotated state, which is calculated by Eq. (15), and *T*_*tRNA*_ is the mean lifetime of the aminoacyl-tRNA bound to the long-paused rotated state, which is calculated by Eq. (11). If the transition from State FS3 to State FS5 occurs (with a probability $P_{3\to 4}$), the remaining mean rotated-state lifetime is calculated by T1'=1/k22+1/k24+1/k25. If the transition from State FS3 to State FS4 occurs (with a probability $1-P_{3\to 4}$), the remaining mean rotated-state lifetime is calculated by T2'=1/k22+1/k26+1/k27+1/k28. As EF-G.GTP binding is the rate-limiting step of these transitions, both T1' and T2' can be approximately calculated by T1'=T2'=1/kb1(G)[EF-G]. Thus, the total mean rotated-state lifetime *T*_*R*3_ approximately has the form

(24) $$T_{R3}=\frac{1}{\left(P_{E}^{\left(1\right)}+P_{LP}^{\left(1\right)}\right)k_{b}^{\left(G\right)}[\text{EF}-G]}+\frac{1-P_{E}^{\left(1\right)}-P_{LP}^{\left(1\right)}}{\left(P_{E}^{\left(1\right)}+P_{LP}^{\left(1\right)}\right)k_{r}}+P_{LP}T',$$

(25) $$T'=N\left(\tau +T_{tRNA}\right)+\frac{1}{k_{b1}^{\left(G\right)}[\text{EF}-G]}$$

With Eqs. (15), (25), Eq. (24) is rewritten as

(26) $$\begin{array}{c} T_{R3}=\left[\frac{1}{k_{b}^{\left(G\right)}\left(P_{E}^{\left(1\right)}+P_{LP}^{\left(1\right)}\right)}+\frac{P_{LP}}{k_{b1}^{\left(G\right)}}\right]\frac{1}{\left[\text{EF}-G\right]}+\frac{1-P_{E}^{\left(1\right)}-P_{LP}^{\left(1\right)}}{P_{E}^{\left(1\right)}+P_{LP}^{\left(1\right)}}\frac{1}{k_{r}}\\ \;+P_{LP}NT_{tRNA}+\frac{P_{LP}N\left(1+T^{\left(G\right)}k_{b1}^{\left(G\right)}[\text{EF}-G]\right)}{k_{b1}^{\left(TC\right)}[\mathrm{TC}]} \end{array}$$

where we show explicitly the dependence of *T*_*R*3_ on [EF-G] and on [TC], while the other parameters are independent of [EF-G] and [TC].

**Fig. 7:**
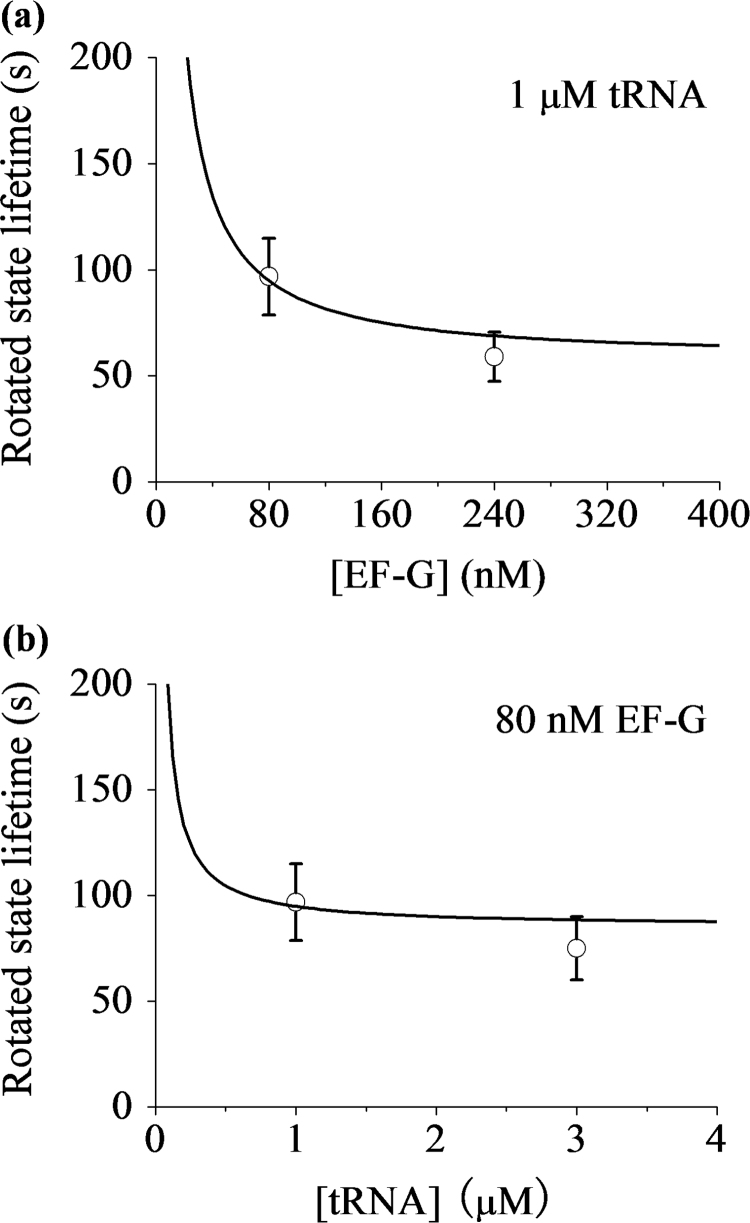
Mean lifetime of the long-paused rotated state occurring at the slippery site. (a) The mean rotated-state lifetime *T*_*R*3_ versus [EF-G] for 1 μM ternary complex (line). The dots are taken from the experimental data (Extended Data Fig. 5d in Chen et al. [29]). (b) The mean rotated-state lifetime *T*_*R*3_ versus the concentration of the cognate ternary complex for 80 nM EF-G (line). The dots are taken from the experimental data (Extended Data Fig. 5e in Chen et al. [29]).

As determined above, we have $k_{b}^{\left(G\right)}$=1.56 ${\mu M}^{-1}s^{-1}$, $k_{b1}^{\left(G\right)}$=0.36 ${\mu M}^{-1}s^{-1}$, $T^{\left(G\right)}k_{b1}^{\left(G\right)}$=0.5 ${\mu M}^{-1}$, $k_{b1}^{\left(TC\right)}$=0.16 ${\mu M}^{-1}s^{-1}$, $P_{LP}^{\left(1\right)}$=0.43 and $P_{E}^{\left(1\right)}$=0.14, and from the experimental data, we have *P*_*LP*_=0.75, *N*=2 and *T*_*tRNA*_=30 s (see Extended Data Fig. 8 in Chen et al. [29]). At low [EF-G] and [TC] as used in the experiments, since the second term in Eq. (26) is much smaller than other terms, the second term can be negligible. With above values, using Eq. (26) the calculated results of *T*_*R*3_ versus [EF-G] and versus [TC] are shown in Fig. 7a and b, respectively. It is interesting to note that without any adjustable parameter, the theoretical data are in good quantitative agreement with the experimental data (see Extended Data Fig. 5d and e in Chen et al. [29]). It is also emphasized that at the slippery site the mean lifetime of the rotated state does not show an inverse dependence on [EF-G] (Fig. 7a), whereas at the non-slippery sites the mean lifetime of the rotated state approximately shows an inverse dependence on [EF-G] [see Eq. (21)]; at the slippery site the mean lifetime of the rotated state is also dependent on [TC], whereas at the non-slippery sites the mean lifetime of the rotated state is independent of [TC] [see Eq. (21)].

**Fig. 8:**
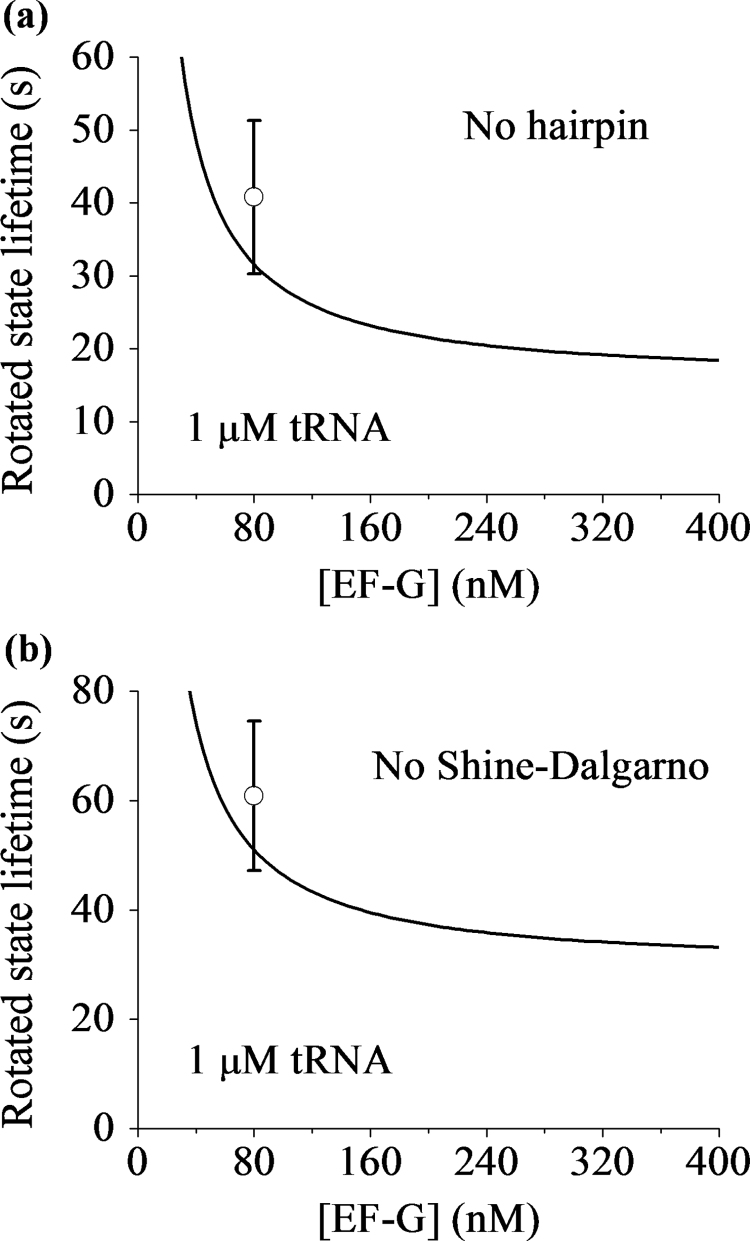
Effects of hairpin and the internal Shine–Dalgarno sequence on the mean lifetime of the long-paused rotated state occurring at the slippery site. (a) The mean rotated-state lifetime *T*_*R*3_ versus [EF-G] for 1 μM ternary complex with no hairpin (line). The dot is taken from the experimental data (Extended Data Fig. 3b in Chen et al. [29]). (b) The mean rotated-state lifetime *T*_*R*3_ versus [EF-G] for 1 μM ternary complex with no internal Shine–Dalgarno sequence (line). The dot is taken from the experimental data (Extended Data Fig. 3d in Chen et al. [29]).

For mutant mRNAs with no hairpin and no internal SD sequence, we can still use Eq. (26) to calculate the mean rotated-state lifetime, with the same values of parameters except *P*_*LP*_, $P_{LP}^{\left(1\right)}$ and $P_{E}^{\left(1\right)}$. With no hairpin and no internal SD sequence, *P*_*LP*_=0.2 and 0.38, respectively (see Extended Data Fig. 3e in Chen et al. [29]). As the resistance to the translocation for the two cases is smaller than the WT case, in our calculation we take $P_{E}^{\left(1\right)}+P_{LP}^{\left(1\right)}$=0.8 that is larger than the WT case. The calculated results of *T*_*R*3_ versus [EF-G] for cases of no hairpin and no internal SD sequence are shown in Fig. 8a and b, respectively. It is noted that the theoretical data are also in quantitative agreement with the experimental data (see Extended Data Fig. 3b and d in Chen et al. [29]).

### 3.6 Mean rotated-state lifetimes of the ribosomes that enter into the long-paused state and the ribosomes that do not enter into the long-paused state

Based on our model (Fig. 3, Fig. 4), at the slippery site (i.e., at codon AAA_24_) where both the SD-antiSD interaction and the downstream hairpin are present, the translating ribosomes either can enter into the long-paused rotated state (transition from State H2 to State LP, with a probability *P*_*LP*_) or cannot enter into the long-paused rotated state (transition from State H2 to State POST, with a probability 1−*P*_*LP*_). Similar to the derivation of Eq. (26), we derive that the mean rotated-state lifetime of the ribosomes that enter into the long-paused state has the form

(27) $$\begin{array}{c} T_{R3}^{\left(LP\right)}=\frac{1}{\left(P_{E}^{\left(1\right)}+P_{LP}^{\left(1\right)}\right)k_{b}^{\left(G\right)}[\text{EF}-G]}+\frac{1-P_{E}^{\left(1\right)}-P_{LP}^{\left(1\right)}}{P_{E}^{\left(1\right)}+P_{LP}^{\left(1\right)}}\frac{1}{k_{r}}\\ +\frac{1}{k_{b1}^{\left(G\right)}[\text{EF}-G]}+NT_{tRNA}+\frac{N\left(1+T^{\left(G\right)}k_{b1}^{\left(G\right)}[\text{EF}-G]\right)}{k_{b1}^{\left(TC\right)}[\mathrm{TC}]} \end{array}$$

The mean rotated-state lifetime of the ribosomes that do not enter into the long-paused state has the form

(28) $$T_{R3}^{\left(NL\right)}=\frac{1}{\left(P_{E}^{\left(1\right)}+P_{LP}^{\left(1\right)}\right)k_{b}^{\left(G\right)}[\text{EF}-G]}+\frac{1-P_{E}^{\left(1\right)}-P_{LP}^{\left(1\right)}}{\left(P_{E}^{\left(1\right)}+P_{LP}^{\left(1\right)}\right)k_{r}}$$

From Eqs. (26), (27), (28), it is noted that $P_{LP}T_{R3}^{\left(LP\right)}+\left(1-P_{LP}\right)T_{R3}^{\left(NL\right)}=T_{R3}$.

**Fig. 9:**
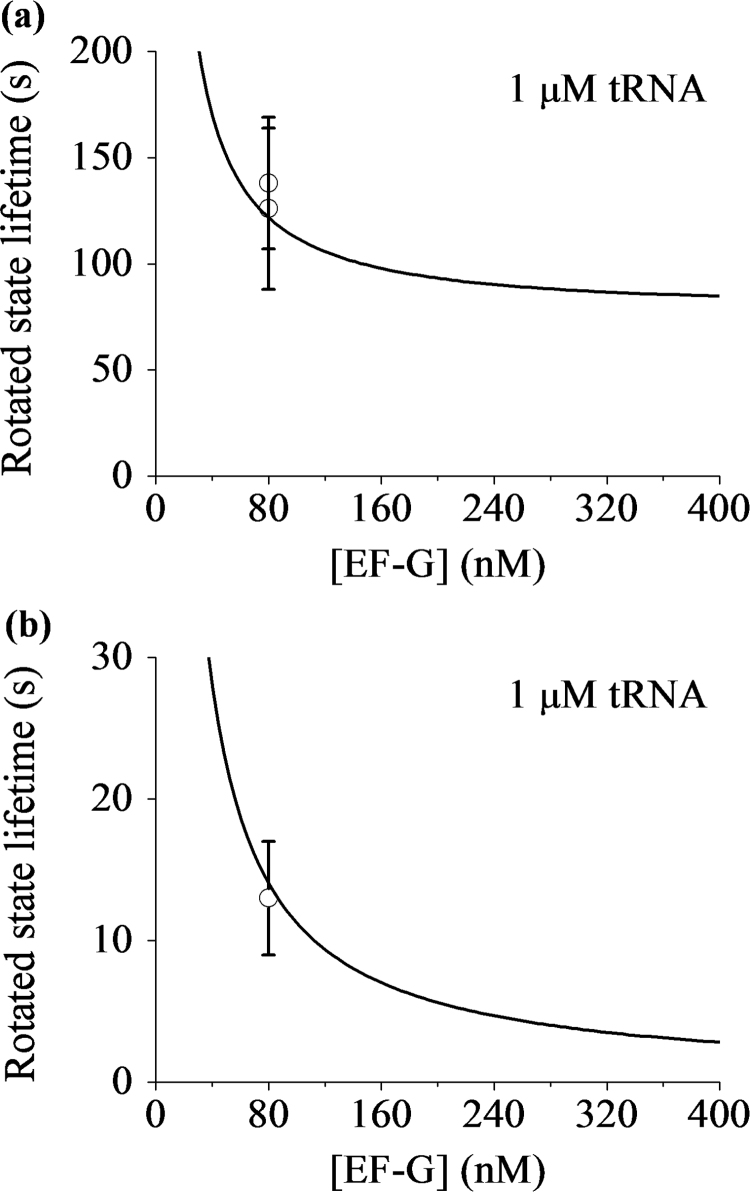
Mean rotated-state lifetimes of the ribosomes that enter into the long-paused state and the ribosomes that do not enter into the long-paused state at the slippery site. (a) The mean rotated-state lifetimes of the ribosomes that enter into the long-paused state versus [EF-G] for 1 μM ternary complex (line). The dots are taken from Chen et al. [29], with one dot being for the case of the WT mRNA and another one for the case of the AAG(AAA) mutant. (b) The mean rotated-state lifetimes of the ribosomes that do not enter into the long-paused state versus [EF-G] for 1 μM ternary complex (line). The dots are taken from Chen et al. [29]. Note that based on our model (Fig. 3) both the WT mRNA and AAG(AAA) mutant give the same *P*_*LP*_ and *P*_*E*_.

With the same values of parameter as those given in the above section (Section 3.5.3) for the WT mRNA, we calculate $T_{R3}^{\left(LP\right)}$ and $T_{R3}^{\left(NL\right)}$ versus [EF-G] by using Eqs. (27), (28). The results are shown in Fig. 9. It is interesting to see that without any adjustable parameter, the theoretical data are also in good quantitative agreement with the three available experimental data at [EF-G]=80 nM [29]. The theoretical data show that both $T_{R3}^{\left(LP\right)}$ and $T_{R3}^{\left(NL\right)}$ decrease with the increase of [EF-G], which can be easily tested by future single-molecule optical trapping assays with different values of [EF-G].

Note that for the WT mRNA, because nearly all the ribosomes that enter into the long-paused rotated state show frameshifing (see Section 3.2), the number of the ribosomes that enter into the long-paused state is equal to that of the ribosomes that frameshift. By contrast, for the mutant mRNAs such as the AAG(AAA) and AAG(UUU), not all the ribosomes that enter into the long-paused rotated state show frameshifing (see Section 3.2) and thus, there are two populations for the non-frameshifted ribosomes: one without the long pause and another one with a long pause that remains in the 0 frame, which is consistent with the experimental data [29].

### 3.7 mRNA translocation is coupled to the intersubunit rotation

It is generally believed that the mRNA translocations in the ribosome, including spontaneous forward and backward translocations [69], EF-G-catalyzed forward translocation [69], EF4-catalyzed forward translocation and EF4-catalyzed backward translocation [69], [70], are coupled to the intersubunit rotations. In other words, the forward translocation is coupled to the reverse intersubunit rotation, while the backward translocation is coupled to the forward intersubunit rotation. Here, we propose that the “uncoupled” translocation that was proposed by Chen et al. [29] in the −1 frameshifting pathway (corresponding to the incomplete +2 translocation in our model of Fig. 3) is in fact also coupled to the reverse intersubunit rotation, as argued by Flanagan et al. [25] that −1 frameshifting is a variant of normal translocation.

### 3.8 Dynamics of translation in the absence of hairpin and the internal SD sequence under the external force

In this section, we consider an mRNA with no hairpin and no internal SD sequence but containing *n* slippery sequences, as shown in Fig. 5b, where only three slippery sequences are drawn. Moreover, we consider an external force, *F*, pulling the mRNA by fixing the body of the 30S subunit. The force can be easily realized by using in vitro optical trapping assays (single trap or dual traps), as described below. In the single-trapping assay, the 30S body of the translating ribosome is fixed to a solid surface, while the end of the downstream mRNA is attached to a micrometer-sized bead held in the trap. Note that the residues on the 30S body which are fixed to the solid surface should be far away from the mRNA channel, so that the fixing has no effect on the translation activity such as the binding of the ternary complex, codon recognition, mRNA translocation and deacylated tRNA dissociation, etc. We can alternatively use the dual-trapping assay, where the 30S body of the translating ribosome is attached to a micrometer-sized bead held in a trap of strong strength, while the end of the downstream mRNA is attached to a micrometer-sized bead held in another trap of weak strength.

Based on our model (Fig. 3), under the external force *F* the occurrence probability of State LP at one slippery sequence which is caused by one reverse intersubunit rotation is calculated by [see Eq. (1)]

(29) $$P_{LP}^{\left(1\right)}=\frac{\exp\left[-\beta \left(\Delta E+2Fp\right)\right]}{\exp\left[-\beta \left(\Delta E+2Fp\right)\right]+\exp\left(-3\beta Fp\right)+\exp\left(-\beta E_{PE}^{\left(50S\right)}\right)},$$

where *p*=0.34 nm. Correspondingly, the occurrence probability of State POST, i.e., the effective translocation, caused by one reverse intersubunit rotation is calculated by [see Eq. (2)]

(30) $$P_{E}^{\left(1\right)}=\frac{\exp\left(-3\beta Fp\right)}{\exp\left[-\beta \left(\Delta E+2Fp\right)\right]+\exp\left(-3\beta Fp\right)+\exp\left(-\beta E_{PE}^{\left(50S\right)}\right)}$$

With Eqs. (29), (30) the occurrence probability of the long-paused rotated state at one slippery sequence is calculated by $P_{LP}=P_{LP}^{\left(1\right)}/\left(P_{LP}^{\left(1\right)}+P_{E}^{\left(1\right)}\right)$, which is rewritten as

(31) $$P_{LP}=\frac{\exp\left[-\beta \left(\Delta E-Fp\right)\right]}{\exp\left[-\beta \left(\Delta E-Fp\right)\right]+1}$$

As shown experimentally [29], when the long-paused state occurs at one slippery sequence shown in Fig. 5b, the occurrence probability of −1 frameshifting is nearly equal to 1. Thus, the total frameshifting probability can be approximately calculated by $P_{FS}=P_{LP}+\left(1-P_{LP}\right)P_{LP}+\cdot \cdot \cdot \cdot \cdot \cdot +{\left(1-P_{LP}\right)}^{n-1}P_{LP}$, which is rewritten as

(32) $$P_{FS}=1-{\left(1-P_{LP}\right)}^{n}$$

**Fig. 10:**
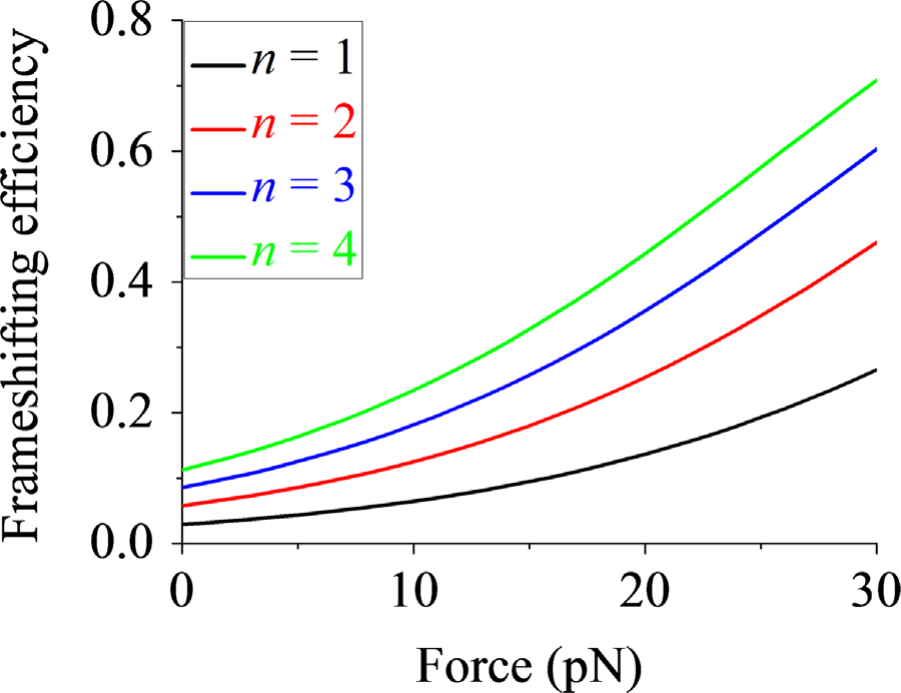
Frameshifting efficiency of translation of the mRNA with no hairpin and no internal Shine–Dalgarno sequence but containing *n* slippery sequences (Fig. 5b) versus the external force acting on the mRNA.

As determined above, we take ΔE=3.5*k*_*B*_*T* here. Using Eqs. (31), (32) we calculate the total frameshifting probability under different force *F* and with different values of *n*. The results of *P*_*FS*_ versus *F* for different values of *n* are shown in Fig. 10. It is seen that at *n*=1 and *F*=0, *P*_*FS*_=0.029, which is consistent with the experimental data of Larsen et al. [18] showing that the efficiency of the *dnaX* slippery sequence alone is about 2% in the cell. The predicted results of *P*_*FS*_ versus *F* for different values of *n* can be easily tested by in vitro experiments as described above.

In the in vitro optical trapping assays as mentioned above, the total frameshifting probability is determined as follows. If no frameshifting occurs the translation would be terminated at the 0 frame stop codon UGA, whereas if a frameshifting occurs the translation would continue after the stop codon. Thus, in the in vitro optical trapping assays, the total frameshifting efficiency is calculated by $P_{FS}=1-P_{Stop}$, where *P*_*Stop*_ is the probability that the translation is terminated at the 0 frame stop codon UGA. Whether the translation is terminated at the stop codon UGA or not can be easily identified by the movement distance of the micrometer-sized bead attached to the mRNA in the optical trapping assays.

### 3.9 Explanations of the experimental data on translocation excursions and broad branching of frameshifting pathways

It is noted that our model can also explain the more recent experimental data that were obtained using the PURExpress in vitro translation system by Yan et al. [30], as discussed below. Based on our model, under large resistance to the forward mRNA translocation arising from the unwinding of the downstream mRNA base pairs and disruption of the upstream SD-antiSD interaction, the codon-anticodon interaction has a large probability to disrupt by reverse intersubunit rotation that occurs after the ribosomal unlocking, as calculations showed before [28]. After the disruption of the codon-anticodon interaction and the SD-antiSD interaction, two scenarios can be expected to occur, with the occurrence probability dependent on the experimental condition. The first and prevalent one in the cell is that the anticodon of the peptidyl-tRNA immediately forms pairing with codon XXX immediately adjacent to codon XXY (see Fig. 3). This leads to entering into State LP, which corresponds to the experiments of Chen et al. [29]. The other scenario is that the anticodon of the peptidyl-tRNA does not rapidly form pairing with another codon that is cognate to the anticodon of the peptidyl-tRNA. The ribosome-tRNA complex then moves randomly relative to the mRNA by the thermal noise until the reformation of the codon-anticodon and SD-antiSD base pairs and then the decoding of the aminoacyl-tRNA in the A site (during the period the ribosome-tRNA complex having the fixed conformation of the non-rotated 30S head and no intersubunit rotation). This scenario corresponds to the experiments that were conducted using the PURExpress in vitro translation system by Yan et al. [30], showing that multiple translocation excursions occur around the slippery sequence, where both the base-pairing interaction of the downstream mRNA and the upstream SD and antiSD interaction are present, and the ribosome can frameshift from various codon positions around the slippery sequence region. Moreover, it is expected that without the codon-anticodon and SD-antiSD interactions the tRNA can dissociate easily from the ribosome, prematurely terminating the translation, which is also consistent with the experimental data of Yan et al. [30]. Note also that without immediate formation of the codon-anticodon interactions, the bending of the peptidyl-tRNA would not occur and thus the long paused state would not occur. This is also consistent with the experimental data of Yan et al. [30].

By contrast, it was proposed by Yan et al. [30] that the multiple translocation excursions are brought by the back-and-forth rotations of the 30S head. This implies that the forward and backward rotations of the 30S head bring about the forward and backward mRNA translocations, respectively, with the forward 30S head rotation bringing about the forward translocation of mRNA coupled with tRNAs to the posttranslocation position while the backward 30S head rotation bringing about the backward movement of the mRNA. It this proposal is true, it would be expected that during the normal translation, no net mRNA movement can occur by a round of the 30S head rotations (the forward and backward rotations) because at the end of a round of the 30S head rotations or in the posttranslocation state both the 30S body and 30S head are in the non-rotated conformations. This is evidently inconsistent with the fact that in the posttranslocation state the mRNA has moved forwards by one codon relative to the pretranslocation state.

## 4 Concluding remarks

We propose a model of the pathway of −1 translational frameshifting during ribosome translation of the *dnaX* −1 frameshift mRNA. In the model, the downstream mRNA base pairs and the upstream SD-antiSD interaction impede the downstream translocation of the 30S subunit along the mRNA, inducing the occurrence of futile translocation besides the usually effective translocation at the non-slippery site. At the slippery site, besides the effective and futile translocations, the incomplete +2 translocation can also occur, inducing the ribosome to enter into a long-paused non-canonical rotated state, where the −1 frameshifting can occur. An important point to note is that the three translocations—the effective translocation, the futile translocation and the incomplete translocation—are coupled with the same reverse intersubunit rotation that occurs after the ribosomal unlocking.

With the model, the single-molecule experimental data on the dynamics of the shunt to either pausing or normal translation, the tRNA transit and sampling dynamics in the long-paused rotated state, the EF-G sampling dynamics, the mean rotated-state lifetimes, etc., are explained quantitatively. The model is also consistent with the experimental data on translocation excursions and broad branching of frameshifting pathways [30]. Moreover, as shown in the next paper [31], with the same model except that the 30S head rotation is included, the biochemical data of Caliskan et al. [32] on the kinetics of EF-G binding and dissociation and on the kinetics of movement of tRNAs inside the ribosome can also be explained quantitatively and well. In addition, we provide predicted results on the dynamics of the −1 frameshifting when translation of the mRNA with no internal SD sequence and no hairpin under the external force on the mRNA. The predicted results can be easily tested by future optical trapping experiments.

Finally, we note that while a major fraction of the −1 frameshifts occur due to the entrance into the long paused state by the incomplete translocation, with a mechanism schematically described in Fig. 3, Fig. 4, it is also possible that a minor fraction of the −1 frameshifts can occur after the effective translocation (State POST, Fig. 3) and before transition to State C0, as analyzed before [28].

## Footnotes

1 Note 1. In the literature, the “non-labile” ribosome is usually called the ribosome in “locking” state. Here, for distinguishing from the “unlocking” state mentioned above, we use the “non-labile” instead of “locking”.

2 Note 2. It is considered that a reverse intersubunit rotation justly makes the 30S subunit move relative to the mRNA by a codon (three nucleotides) in the 3′ direction with the 50S subunit being kept fixed to the two tRNAs (the effective translocation) or make the 50S subunit move relative to the mRNA by three nucleotides in the 5′ direction (i.e., the two tRNAs move from the 50S E and P sites to 50S P and A sites) but with the 30S subunit being kept fixed to the mRNA (the futile translocation). Thus, the two cases will not cause bending of the tRNAs. However, if the reverse intersubunit rotation is realized by making 30S subunit move relative to the mRNA by two nucleotides in the 3′ direction and making 50S subunit move relative to the mRNA by one nucleotide in the 5′ direction, considering that the peptidyl moiety is fixed to the 50S P site by the specific interaction between them and the anticodon of the peptidyl-tRNA is paring with codon XXX, the movements would cause bending of the peptidyl-tRNA.

Appendix A Supplementary data associated with this article can be found in the online version at http://dx.doi.org/10.1016/j.bbrep.2016.01.017.

## References

[1] Jacks T., Power M.D., Masiarz F.R., Luciw P.A., Barr P.J., Varmus H.E. (1988). Characterization of ribosomal frameshifting in HIV-1 *gag*-*pol* expression. Nature.

[2] Vaishnav Y.N., Wong-Staal F. (1991). The biochemistry of AIDS. Annu. Rev. Biochem..

[3] Baril M., Dulude D., Gendron K., Lemay G., Brakier-Gingras L. (2003). Efficiency of a programmed –1 ribosomal frameshift in the different subtypes of the human immunodeficiency virus type 1 group M. RNA.

[4] Biswas P., Jiang X., Pacchia A.L., Dougherty J.P., Peltz S.W. (2004). The human immunodeficiency virus type 1 ribosomal frameshifting site is an invariant sequence determinant and an important target for antiviral therapy. J. Virol..

[5] Plant E.P., Dinman J.D. (2008). The role of programmed –1 ribosomal frameshifting in coronavirus propagation. Front. Biosci..

[6] Marcheschi R.J., Tonelli M., Kumar A., Butcher S.E. (2011). Structure of the HIV-1 frameshift site RNA bound to a small molecule inhibitor of viral replication. ACS Chem. Biol..

[7] Wang M., Wang Y., Sun X., Cheng J., Fu Y., Liu H., Jiang D., Ghabrial S.A., Xie J. (2015). Characterization of a novel megabirnavirus from *Sclerotinia sclerotiorum* reveals horizontal gene transfer from single-stranded RNA virus to double-stranded RNA virus. J. Virol..

[8] Farabaugh P.J. (1996). Programmed translational frameshifting. Microbiol. Rev..

[9] Dinman J.D. (2012). Mechanisms and implications of programmed translational frameshifting. Wiley Interdiscip. Rev. RNA.

[10] Harger J.W., Meskauskas A., Dinman J.D. (2002). An “integrated model” of programmed ribosomal frameshifting. Trends Biochem. Sci..

[11] Dunkle J.A., Dunham C.M. (2015). Mechanisms of mRNA frame maintenance and its subversion during translation of the genetic code. Biochimie.

[12] Liao P.Y., Gupta P., Petrov A.N., Dinman J.D., Lee K.H. (2008). A new kinetic model reveals the synergistic effect of E-, P- and A-sites on +1 ribosomal frameshifting. Nucleic Acids Res..

[13] Xie P. (2014). Dynamics of +1 ribosomal frameshifting. Math. Biosci..

[14] Weiss R.B., Dunn D.M., Shuh M., Atkins J.F., Gesteland R.F., Coli E. (1989). Ribosomes re-phase on retroviral frameshift signals at rates ranging from 2% to 50%. New Biol..

[15] Dinman J.D., Icho T., Wickner R.B. (1991). A −1 ribosomal frameshift in a double-stranded RNA virus of yeast forms a *gag*-*pol* fusion protein. Proc. Natl. Acad. Sci. USA.

[16] Brierley I., Jenner A.J., Inglis S.C. (1992). Mutational analysis of the “slippery-sequence” component of a coronavirus ribosomal frameshifting signal. J. Mol. Biol..

[17] Giedroc D.P., Cornish P.V. (2009). Frameshifting RNA pseudoknots: structure and mechanism. Virus Res..

[18] Larsen B., Wills N.M., Gesteland R.F., Atkins J.F. (1994). rRNA-mRNA base pairing stimulates a programmed −1 ribosomal frameshift. J. Bacteriol..

[19] Tsuchihashi Z., Kornberg A. (1990). Translational frameshifting generates the gamma subunit of DNA polymerase III holoenzyme. Proc. Natl. Acad. Sci. USA.

[20] Tsuchihashi Z., Brown P.O. (1992). Sequence requirements for efficient translational frameshifting in the Escherichia coli dnaX gene and the role of an unstable interaction between tRNA(Lys) and an AAG lysine codon. Genes Dev..

[21] Larsen B., Gesteland R.F., Atkins J.F. (1997). Structural probing and mutagenic analysis of the stem-loop required for Escherichia coli dnaX ribosomal frameshifting: programmed efficiency of 50%. J. Mol. Biol..

[22] Leger M., Dulude D., Steinberg S.V., Brakier-Gingras L. (2007). The three transfer RNAs occupying the A, P and E sites on the ribosome are involved in viral programmed −1 ribosomal frameshift. Nucleic Acids Res..

[23] Plant E.P., Jacobs K.L.M., Harger J.W., Meskauskas A., Jacobs J.L., Baxter J.L., Petrov A.N., Dinman J.D. (2003). The 9-Å solution: how mRNA pseudoknots promote efficient programmed −1 ribosomal frameshifting. RNA.

[24] Namy O., Moran S.J., Stuart D.I., Gilbert R.J., Brierley I. (2006). A mechanical explanation of RNA pseudoknot function in programmed ribosomal frameshifting. Nature.

[25] Flanagan J.F., Namy O., Brierley I., Gilbert R.J.C. (2010). Direct observation of distinct A/P hybrid-state tRNAs in translocating ribosomes. Structure.

[26] Liao P.-Y., Choi Y.S., Dinman J.D., Lee K.L. (2011). The many paths to frameshifting: kinetic modeling and analysis of the effects of different elongation steps on programmed −1 ribosomal frameshifting. Nucleic Acids Res..

[27] Bailey B.L., Visscher K., Watkins J. (2014). A stochastic model of translation with −1 programmed ribosomal frameshifting. Phys. Biol..

[28] Xie P. (2013). A dynamical model of programmed −1 ribosomal frameshifting. J. Theor. Biol..

[29] Chen J., Petrov A., Johansson M., Tsai A., O’Leary S.E., Puglisi J.D. (2014). Dynamic pathways of −1 translational frameshifting. Nature.

[30] Yan S., Wen J.-D., Bustamante C., Tinoco I. (2015). Ribosome excursions during mRNA translocation mediate broad branching of frameshift pathways. Cell.

[31] P. Xie, Model of the pathway of –1 frameshifting: kinetics. Submitted to Biochem. Biophys. Rep,, 2016. 10.1016/j.bbrep.2016.02.008 PMC5600437 28955853

[32] Caliskan N., Katunin V.I., Belardinelli R., Peske F., Rodnina M.V. (2014). Programmed –1 frameshifting by kinetic partitioning during impeded translocation. Cell.

[33] Demeshkina N., Jenner L., Westhof E., Yusupov M., Yusupova G. (2012). A new understanding of the decoding principle on the ribosome. Nature.

[34] Moazed D., Noller H.F. (1989). Intermediate states in the movement of transfer RNA in the ribosome. Nature.

[35] Zavialov A.V., Ehrenberg M. (2003). Peptidyl-tRNA regulates the GTPase activity of translation factors. Cell.

[36] Valle M., Zavialov A., Sengupta J., Rawat U., Ehrenberg M., Frank J. (2003). Locking and unlocking of ribosomal motions. Cell.

[37] Blanchard S.C., Kim H.D., Gonzalez R.L., Puglisi J.D., Chu. S. (2004). tRNA dynamics on the ribosome during translation. Proc. Natl Acad. Sci. USA.

[38] Fei J., Kosuri P., MacDougall D.D., Gonzalez R.L. (2008). Coupling of ribosomal L1 stalk and tRNA dynamics during translation elongation. Mol. Cell.

[39] Cornish P.V., Ermolenko D.N., Noller H.F., Ha T. (2008). Spontaneous intersubunit rotation in single ribosomes. Mol. Cell.

[40] Zavialov A.V., Hauryliuk V.V., Ehrenberg M. (2005). Guanine-nucleotide exchange on ribosome-bound elongation factor G initiates the translocation of tRNAs. J. Biol..

[41] Spiegel P.C., Ermolenko D.N., Noller H.F. (2007). Elongation factor G stabilizes the hybrid-state conformation of the 70S ribosome. RNA.

[42] Joseph S., Noller H.F. (1998). EF-G–catalyzed translocation of anticodon stem–loop analogs of transfer RNA in the ribosome. EMBO J..

[43] Savelsbergh A., Katunin V.I., Mohr D., Peske F., Rodnina M.V., Wintermeyer W. (2003). An elongation factor G-induced ribosome rearrangement precedes tRNA-mRNA translocation. Mol. Cell.

[44] Lill R., Robertson J.M., Wintermeyer W. (1989). Binding of the 30-terminus of tRNA to 23S rRNA in the ribosomal exit site actively promotes translocation. EMBO J..

[45] Feinberg J.S., Joseph S. (2001). Identification of molecular interactions between P-site tRNA and the ribosome essential for translocation. Proc. Natl Acad. Sci. USA.

[46] Frank J., Agrawal R.K. (2000). A ratchet-like inter-subunit reorganization of the ribosome during translocation. Nature.

[47] Xie P. (2015). Model of ribosomal translocation coupled with intra- and inter-subunit rotations. Biochem. Biophys. Rep..

[48] Xie P. (2014). Biphasic character of ribosomal translocation and non-Michaelis-Menten kinetics of translation. Phys. Rev. E.

[49] Chen C., Stevens B., Kaur J., Cabral D., Liu H., Wang Y., Zhang H., Rosenblum G., Smilansky Z., Goldman Y.E., Cooperman B. (2011). Single-molecule fluorescence measurements of ribosomal translocation dynamics. Mol. Cell.

[50] Chen J., Petrov A., Tsai A., O’Leary S.E., Puglisi J.D. (2013). Coordinated conformational and compositional dynamics drive ribosome translocation. Nat. Struct. Mol. Biol..

[51] Xie P. (2013). Translocation dynamics of tRNA-mRNA in the ribosome. Biophys. Chem..

[52] Lin J., Gagnon M.G., Bulkley D., Steitz T.A. (2015). Conformational changes of elongation factor G on the ribosome during tRNA translocation. Cell.

[53] Xie P. (2014). An explanation of biphasic characters of mRNA translocation in the ribosome. BioSystems.

[54] Pan D., Kirillov S.V., Cooperman B.S. (2007). Kinetically competent intermediates in the translocation step of protein synthesis. Mol. Cell.

[55] Xie P. (2013). Model of ribosome translation and mRNA unwinding. Eur. Biophys. J..

[56] Qu X., Wen J.-D., Lancaster L., Noller H.F., Bustamante C., Tinoco I. (2011). The ribosomeuses two activemechanisms to unwind messenger RNA during translation. Nature.

[57] Wen J.-D., Lancaster L., Hodges C., Zeri A.-C., Yoshimura S.H., Noller H.F., Bustamante C., Tinoco I. (2008). Following translation by single ribosomes one codon at a time. Nature.

[58] Chen C., Zhang H., Broitman S.L., Reiche M., Farrell I., Cooperman B.S., Goldman Y.E. (2013). Dynamics of translation by single ribosomes through mRNA secondary structures. Nat. Struct. Mol. Biol..

[59] Xie P. (2015). Dwell-time distribution, long pausing and arrest of single-ribosome translation through the mRNA duplex. Int. J. Mol. Sci..

[60] Xie P. (2014). Dynamics of tRNA translocation, mRNA translocation and tRNA dissociation during ribosome translation through mRNA secondary structures. Eur. Biophys. J..

[61] Kim H.-K., Liu F., Fei J., Bustamante C., Gonzalez R.L., Tinoco I. (2014). A frameshifting stimulatory stem loop destabilizes the hybrid state and impedes ribosomal translocation. Proc. Natl Acad. Sci. USA.

[62] Xie P. (2014). Origin of multiple intersubunit rotations before EF-G-catalyzed ribosomal translocation through the mRNA with a downstream secondary structure. BMC Biophys..

[63] Rodnina M.V., Gromadski K.B., Kothe U., Wieden H.-J. (2005). Recognition and selection of tRNA in translation. FEBS Lett..

[64] Pape T., Wintermeyer W., Rodnina M.V. (1999). Induced fit in initial selection and proofreading of aminoacyl-tRNA on the ribosome. EMBO J..

[65] Freier S.M., Kierzek R., Jaeger J.A., Sugimoto N., Caruthers M.H., Neilson T., Tuener D.H. (1986). Improved free-energy parameters for predictions of RNA duplex stability. Proc. Natl Acad. Sci. USA.

[66] Xie P. (2013). Dynamics of tRNA occupancy and dissociation during translation by the ribosome. J. Theor. Biol..

[67] Wintermeyer W., Peske F., Beringer M., Gromadski K.B., Savelsbergh A., Rodnina M.V. (2004). Mechanisms of elongation on the ribosome: dynamics of a macromolecular machine. Biochem. Soc. Trans..

[68] Kim H.D., Puglisi J., Chu S. (2007). Fluctuations of tRNAs between classical and hybrid states. Biophys. J..

[69] Xie P. (2013). Dynamics of forward and backward translocation of mRNA in the ribosome. PLoS One.

[70] Xie P. (2014). Model of EF4-induced ribosomal state transitions and mRNA translocation. Phys. Biol..

