# Supplementary material for: Model of the pathway of −1 frameshifting: Long pausing
Source: Biochem Biophys Rep. 2016 Jan 29;5:408–24. doi: 10.1016/j.bbrep.2016.01.017 (PMC5600365; doi:10.1016/j.bbrep.2016.01.017)
Supplement: Supplementary file 2 — Supplementary material [file mmc2.pdf]

Supplementary Material

Supplementary Text

S1. The probability of the normal translocation that is calculated based on pathway of Fig. S1 is not in quantitative agreement with the experimental data

As in Chen et al. (2014), we consider only two conformations of mRNA duplex: open conformation, in which three RNA base pairs immediate in front of the mRNA entry site of the ribosome are open, and closed conformation, in which no base pair immediate in front of the mRNA entry site is open, i.e., all base pairs in front of the ribosome are closed. The probability of the mRNA duplex in the open conformation can be calculated by

$$f_O = \frac{\exp\left(-3\beta E_{bp}\right)}{1+\exp\left(-3\beta E_{bp}\right)}, \quad \text{(S1)}$$

where E_{bp} is the free energy change of unwinding one mRNA base pair (G:C) and $\beta^{-1} = k_B T$, with k_B the Boltzmann constant and T the absolute temperature. The probability of the mRNA duplex in the closed conformation then has the form

$$f_C = 1 - f_O = \frac{1}{1+\exp\left(-3\beta E_{bp}\right)}. \quad \text{(S2)}$$

As estimated in the main text (see Section 3.1), the free energy change of unwinding one mRNA base pair is about $2.5k_BT$. Then, with Eq. (S1) we have f_O = 5.5×10^{-4}. This implies that in the pathway of Fig. S1, even without the formation of the Shine–Dalgarno and anti-Shine–Dalgarno (SD:anti-SD) pairings, the probability of the normal translocation is only 5.5×10^{-4}, which significantly deviates from the single-molecule experimental data of about 25% (Chen et al., 2014). Conversely, in order to be consistent with the experimental data of f_O = 25%, from Eq. (S1) we obtain E_{bp} = $0.367k_BT$= 0.217 kcal/mol, which is a too small value to be unreasonable.

From our calculation it is seen that the probability of the mRNA duplex in the

open conformation is only 5.5×10^{-4}, implying that the mRNA duplex is in fact always in the closed conformation. Then, it is difficult to understand why a fraction of the normal translocations (25%) occur and another fraction of the "uncoupled" translocations (75%) occur. Moreover, as the "uncoupled" translocation involves no reverse intersubunit rotation, then how the "uncoupled" translocation occurs is unclear. In addition, since after EF-G release from State R2 (Fig. S1) the state becomes similar to State R5 (Fig. S1), why EF-G.GTP cannot bind to State R2 and then catalyzes the reverse intersubunit rotation whereas EF-G.GTP can bind to State R5 and then catalyzes the reverse intersubunit rotation is not clear.

S2. Probability of effective translocation during translation of each codon on the *dnaX* mRNA

Available experimental data (Chen et al., 2013; Takyar et al., 2005; Qu et al., 2011) and analyses (Xie, 2014a; 2014b) indicated that the 30S subunit of the ribosome covers about 12 bases (four codons) of the single-stranded mRNA from its P site to the mRNA entry site. Thus, when the 30S P site is occupying GCA_{21} codon of the wild-type mRNA used in the experiments of Chen et al. (2014), the G_{34}:C base pair is just outside the mRNA entry site (see Fig. S2b); and when the 30S P site is occupying AAA_{24} codon, three G:C base pairs in the mRNA stem loop are unwound (see Fig. S2c). Moreover, it is considered that when the 30S P site is occupying GCA_{21} codon, the anti-Shine-Dalgarno (anti-SD) of 16S rRNA is in the same position of the Shine-Dalgarno (SD) sequence of the mRNA along the translation direction (see Fig. S2b). Under this consideration, we study the probability of the effective translocation during translation of each codon on the mRNA.

(i). During the translation of codons before codon GCA_{21}, no downstream mRNA base pair is required to unwind during the translocation step and moreover, since the anti-SD is far away from the SD, there is no the SD:anti-SD interaction. Thus, no resistance is present to hinder the translocation, as during the translation through the single-stranded mRNA, giving the probability of effective translocation, $P_E^{(1)} = 1$.

(ii). During the translation of codon GCA_{21}, the translocation (from Fig. S2a to S2b) also requires no unwinding of mRNA base pair. Moreover, the SD:anti-SD

interaction facilitates the translocation. Thus, the reverse intersubunit rotation gives a 100% probability of the effective translocation.

(iii). During the translation of codon AAA_{24}, the translocation (from Fig. S2b to S2c) requires unwinding of three G:C base pairs, giving an increase in free energy of $3E_{bp}$, and moreover, due to the SD:anti-SD interaction the translocation (from Fig. S2b to S2c) also gives an increase in free energy of $\Delta E_{SD}^{(3)}$ (defined in Section 3.1). Thus, the effective-translocation probability $P_E^{(1)}$ can be calculated by

$$P_E^{(1)} = \frac{\exp\left(-\frac{\Delta E_{SD}^{(3)} + 3E_{bp}}{k_B T}\right)}{\exp\left(-\frac{\Delta E_{SD}^{(3)} + 3E_{bp}}{k_B T}\right) + \exp\left(-\frac{E_{PE}^{(50S)}}{k_B T}\right)}. \quad \text{(S2)}$$

Eq. (S2) is the same as Eq. (19). As shown in Section 3.4, we have $P_E^{(1)} = 0.25$.

(iv). During the translation of codon AAG_{27}, the translocation (from Fig. S2c to S2d) requires unwinding of three G:C base pairs, giving an increase in free energy of $3E_{bp}$. Moreover, since the anti-SD is far away from the SD sequence, the SD:anti-SD interaction can be negligible. Thus, the effective-translocation probability $P_E^{(1)}$ can be calculated by

$$P_E^{(1)} = \frac{\exp\left(-\frac{3E_{bp}}{k_B T}\right)}{\exp\left(-\frac{3E_{bp}}{k_B T}\right) + \exp\left(-\frac{E_{PE}^{(50S)}}{k_B T}\right)}. \quad \text{(S3)}$$

As determined in Section 3.1, $E_{bp} = 2.5k_BT$. As determined previously (Xie, 2013), $E_{PE}^{(50S)} = 9k_BT$. With these values, using Eq. (1) we obtain $P_E^{(1)} = 0.82$. Comparing Eq. (22) with Eq. (24), it is seen that even with the second term of Eq. (24) being negligibly smaller than the first term, this $P_E^{(1)} = 0.82$ that is slightly smaller than 1 gives the mean rotated-state lifetime T_{R2} to be slightly larger than T_{R1}, which is consistent with the experimental data [see, e.g., Figure 1d and Extended Data Figure 5c – f in Chen et al. (2014)].

(v). During the translation of codon AGU_{30}, the translocation requires unwinding of two U:A base pairs and one G:C base pair, giving an increase in free energy of $2E_{bp}^{(U:A)} + E_{bp}$, where $E_{bp}^{(U:A)}$ is the base-pairing energy of a U:A base pair and E_{bp} is

the base-pairing energy of a G:C base pair. Moreover, since the anti-SD is far away from the SD sequence, the SD:anti-SD interaction can be negligible. Thus, the effective-translocation probability $P_E^{(1)}$ can be calculated by

$$P_E^{(1)} = \frac{\exp\left(-\dfrac{2E_{bp}^{(U:A)} + E_{bp}}{k_B T}\right)}{\exp\left(-\dfrac{2E_{bp}^{(U:A)} + E_{bp}}{k_B T}\right) + \exp\left(-\dfrac{E_{PE}^{(50S)}}{k_B T}\right)}, \quad \text{(S3)}$$

As mentioned above, E_{bp} = $2.5k_BT$ and $E_{PE}^{(50S)}$ = $9k_BT$. As the base-pairing energy of a U:A base pair is about half of that of a G:C base pair (Freier et al., 1986), we take $E_{bp}^{(U:A)} = E_{bp}/2$. With these values, using Eq. (S3) we obtain $P_E^{(1)}$ = 0.982, i.e., $P_E^{(1)} \approx 1$.

(vi). During the translation of codon GAU_{33}, only three isolated G:C base pairs exist, which can be easily opened by the thermal noise. Thus, the translocation is similar to that of translation through the single-stranded mRNA, giving the probability of the effective translocation, $P_E^{(1)} \approx 1$.

(vii). After the translation of codon GAU_{33}, no mRNA base pair is present. Thus, during the translation of the remaining codons the probability of effective translocation, $P_E^{(1)}$ = 1.

References

Chen C., Zhang H., Broitman S.L., Reiche M., Farrell I., Cooperman B.S., Goldman Y.E. (2013) Dynamics of translation by single ribosomes through mRNA secondary structures. Nature Struct. Mol. Biol. 20, 582–588.

Chen J., Petrov A., Johansson M., Tsai A., O'Leary S.E., Puglisi J.D. (2014) Dynamic pathways of –1 translational frameshifting. Nature 512, 328–332.

Freier S.M., Kierzek R., Jaeger J.A., Sugimoto N., Caruthers M.H., Neilson T., Tuener D.H. (1986) Improved free-energy parameters for predictions of RNA duplex stability. Proc. Natl Acad. Sci. USA 83, 9373–9377.

Qu X., Wen J.-D., Lancaster L., Noller H.F., Bustamante C., Tinoco Jr I. (2011) The ribosomeuses two activemechanisms to unwind messenger RNA during translation. Nature 475, 118–121.

Takyar S., Hickerson R.P., Noller H.F. (2005) mRNA helicase activity of the ribosome. Cell 120, 49–58.

Xie P. (2013) Model of ribosome translation and mRNA unwinding. Eur. Biophys. J. 42, 347–354.

Xie P. (2014a) Dynamics of tRNA translocation, mRNA translocation and tRNA dissociation during ribosome translation through mRNA secondary structures. Eur. Biophys. J. 43, 229–240.

Xie P. (2014b) On the mechanical force generated by EF-G-catalyzed ribosome translocation. J. Mol. Biochem. 3, 58-63.

Supplementary Figures

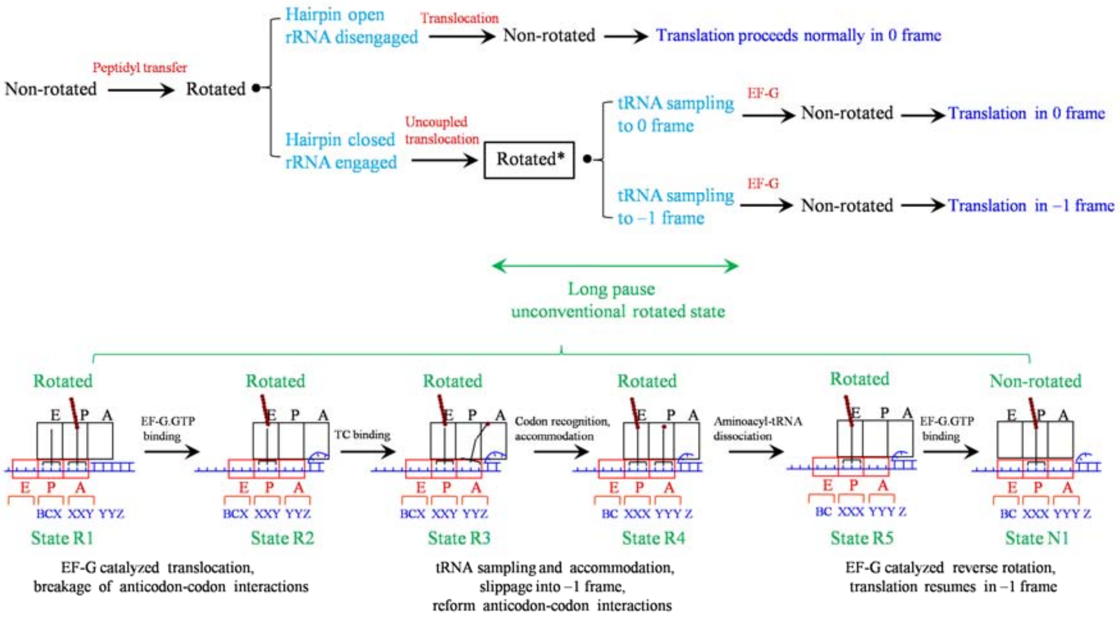

Fig. S1. Schematic representation of the branchpoint of pathways and mechanism of *dnaX* –1 frameshifting proposed by Chen et al. (2014). The figure is redrawn from Chen et al. (2014). The first branchpoint during frameshifting is probably due to the stochastic interaction of the ribosome with the hairpin in an open or closed state, and/or formation of the Shine–Dalgarno and anti-Shine–Dalgarno pairing, that represent the shunt to either pausing or normal translation. If the hairpin is in open state, the translocation of the ribosomes with normal translation is coupled with the reverse intersubunit rotation, leading to +3 translocation. If the hairpin is in closed state, the translocation of the paused ribosomes is uncoupled with the reverse intersubunit rotation (called "uncoupled" translocation), leading also to +3 translocation and creating a non-canonical intermediate in translation (denoted Rotated*). The "uncoupled" translocation exposes the A site, to which $tRNA^{Lys}$ and EF-G sample. $tRNA^{Lys}$ sampling and accommodation to the AAG codon and EF-G action stimulates the ribosome to slip into the –1 frame. Finally, EF-G catalyses the final reverse intersubunit rotation, after which the ribosome resumes normal translation.

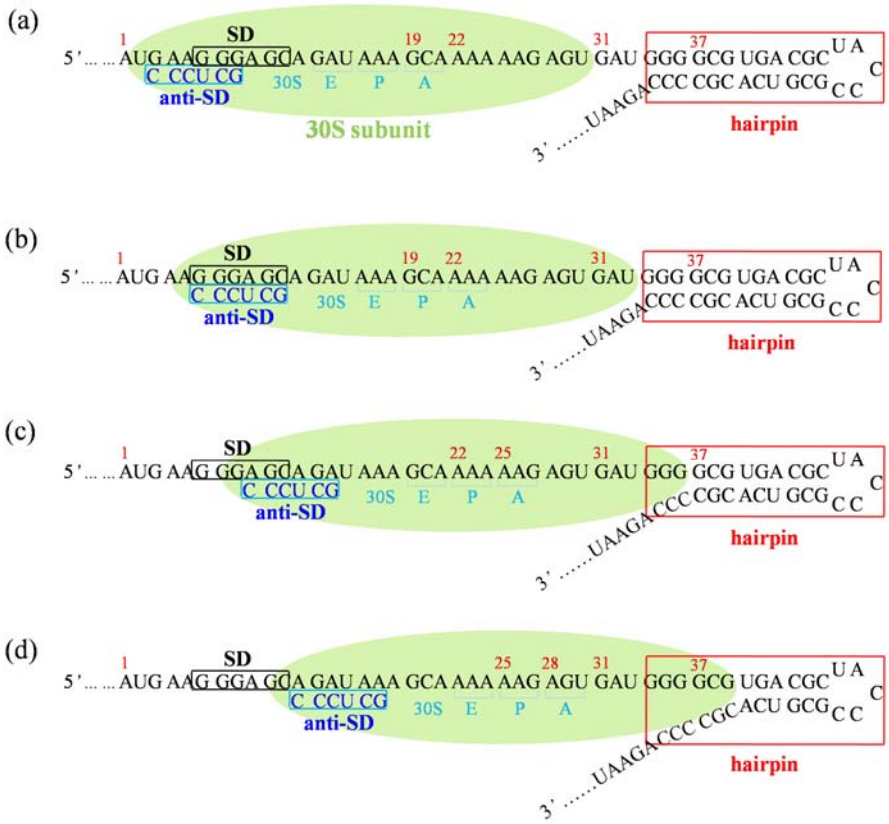

Fig. S2. Schematic representation of the positions of the ribosome on the wild type mRNA used in Chen et al. (2014). The Shine–Dalgarno sequence (SD) and the hairpin in the mRNA are indicated by a black block and a red block, respectively. The green oval represents the 30S subunit of the ribosome, with the 30S A, P and E sites and the anti-Shine–Dalgarno (anti-SD) being indicated. The ribosome covers 12 bases (or four codons) of the single-stranded mRNA from its P site to the mRNA entry site. **(a)** The posttranslocation state during translation of codon AAA_{18} or the pretranslocation state during translation of codon GCA_{21}. No mRNA base pair in the hairpin is unwound. **(b)** The posttranslocation state during translation of codon GCA_{21} or the pretranslocation state during translation of codon AAA_{24}. No mRNA base pair in the hairpin is unwound. **(c)** The posttranslocation state during translation of codon AAA_{24} or the pretranslocation state during translation of codon AAG_{27}. Three mRNA base pairs in the hairpin are unwound. **(d)** The posttranslocation state during translation of codon AAG_{27} or the pretranslocation state during translation of codon AGU_{30}. Six mRNA base pairs in the hairpin are unwound.
